# High-quality genome assembly and resequencing of modern cotton cultivars provide resources for crop improvement

**DOI:** 10.1038/s41588-021-00910-2

**Published:** 2021-08-09

**Authors:** Zhiying Ma, Yan Zhang, Liqiang Wu, Guiyin Zhang, Zhengwen Sun, Zhikun Li, Yafei Jiang, Huifeng Ke, Bin Chen, Zhengwen Liu, Qishen Gu, Zhicheng Wang, Guoning Wang, Jun Yang, Jinhua Wu, Yuanyuan Yan, Chengsheng Meng, Lihua Li, Xiuxin Li, Shaojing Mo, Nan Wu, Limei Ma, Liting Chen, Man Zhang, Aijun Si, Zhanwu Yang, Nan Wang, Lizhu Wu, Dongmei Zhang, Yanru Cui, Jing Cui, Xing Lv, Yang Li, Rongkang Shi, Yihong Duan, Shilin Tian, Xingfen Wang

**Affiliations:** 1 State Key Laboratory of North China Crop Improvement and Regulation, Key Laboratory for Crop Germplasm Resources of Hebei, Hebei Agricultural University, Baoding, China; 2 Novogene Bioinformatics Institute, Beijing, China

**Keywords:** DNA sequencing, Sequence annotation, DNA sequencing, Sequence annotation, Genome-wide association studies

## Abstract

Cotton produces natural fiber for the textile industry. The genetic effects of genomic structural variations underlying agronomic traits remain unclear. Here, we generate two high-quality genomes of *Gossypium hirsutum* cv. NDM8 and *Gossypium barbadense* acc. Pima90, and identify large-scale structural variations in the two species and 1,081 G*. hirsutum* accessions. The density of structural variations is higher in the D-subgenome than in the A-subgenome, indicating that the D-subgenome undergoes stronger selection during species formation and variety development. Many structural variations in genes and/or regulatory regions potentially influencing agronomic traits were discovered. Of 446 significantly associated structural variations, those for fiber quality and Verticillium wilt resistance are located mainly in the D-subgenome and those for yield mainly in the A-subgenome. Our research provides insight into the role of structural variations in genotype-to-phenotype relationships and their potential utility in crop improvement.

## Abstract (web summary)

High-quality genomes of two cultivated tetraploid cottons *Gossypium hirsutum* cv. NDM8 and *Gossypium barbadense* acc. Pima90 and resequencing of 1,081 *G. hirsutum* accessions provide insights into the role of structural variations.

## Main

As a widely cultivated fiber crop, cotton produces natural fiber for the textile industry^1^. *G.* *hirsutum* accounts for more than 90% of the yield in production. Thousands of improved cotton varieties have played pivotal roles in yield increases^2^. On this basis, breeders strive to create new varieties by synergistically increasing genetically complex yield and quality while obtaining resistance to numerous adversities, which is limited, however, by insufficient knowledge and understanding of the genomic basis of key agronomic traits^3^. High-quality genome assembly for modern *G.* *hirsutum* varieties, as well as for obsolete varieties TM-1 and ZM24 (refs. ^4–6^), is crucial to breeding and biology research; however, genomic information in recently developed cottons has been limited, and genomic diversification in modern breeding process remains unclear.

*G.* *barbadense* occupies roughly 10% of the yield and affords high-quality lint fibers. To improve the fibers and disease resistance of *G.* *hirsutum*, a proposed approach is to transfer superior related traits from *G.* *barbadense* into *G.* *hirsutum*; however, genomic variations in *G.* *barbadense* compared with modern *G. hirsutum* are not clear. The identification of associated single nucleotide polymorphisms (SNPs) increases understanding of the genetic basis of cotton agricultural traits^2,7,8^. Widespread genomic structural variations, generally defined as insertion, deletion, inversion and translocation, mean that any single haplotype may be missing or contain sequence variants that are not present in most of the population^9,10^. Therefore, exploring structural variations is imperative for cotton improvement on the basis of genome assemblies and resequencing data from more accessions. Meanwhile, the genetic effects of structural variations underlying traits are less known.

In this study, we generated two high-quality reference genomes and annotations for the modern *G.* *hirsutum* cv. NDM8 and *G.* *barbadense* acc. Pima90. NDM8 is widely grown in Yellow River Valley cotton-producing areas of China, and Pima90 has served as a genetic material in molecular breeding^11–16^. Furthermore, we resequenced 1,081 worldwide *G.* *hirsutum* accessions, consisting of a core collection^8^ plus some modern and obsolete varieties with disease resistance and glandlessness. Analyzing the two genomes and resequences showed that large-scale genomic variations occurred during breeding, providing resources for cotton crop improvement.

## Results

### High-quality genomes of tetraploid cottons NDM8 and Pima90

**Extended Data Fig. 1:**
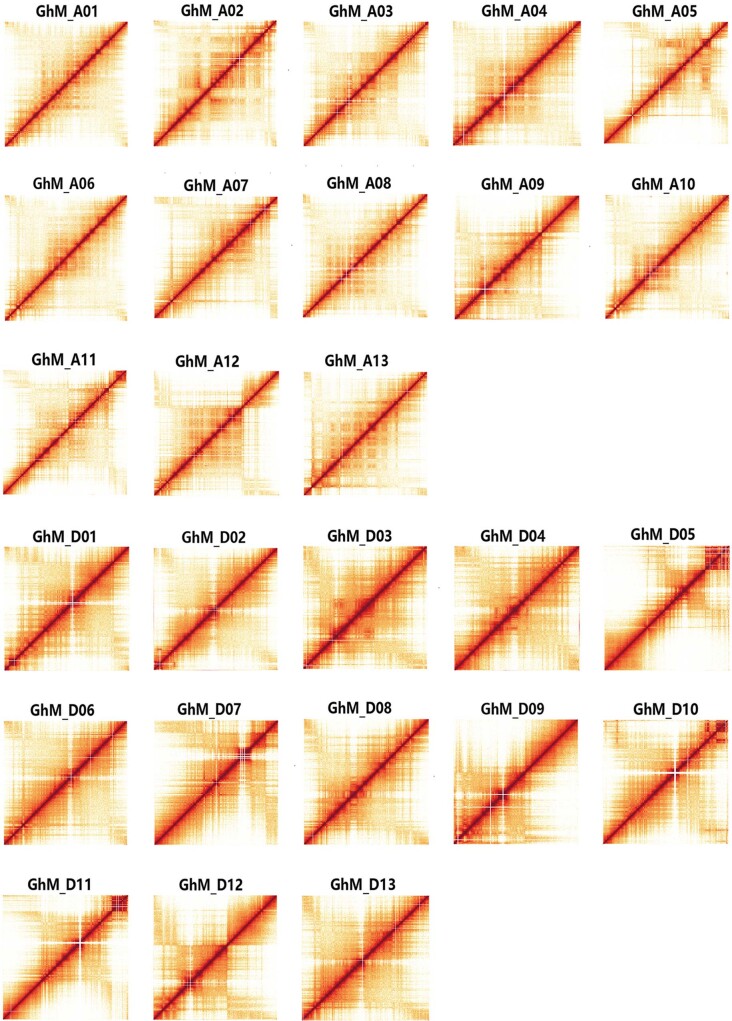
Chromatin interactions in each chromosome of *G. hirsutum* NDM8. Each heatmap is shown at a resolution of 100 kb. The dark red dots show the high probability of interaction, and the light dots show the low probability of interaction.

**Extended Data Fig. 2:**
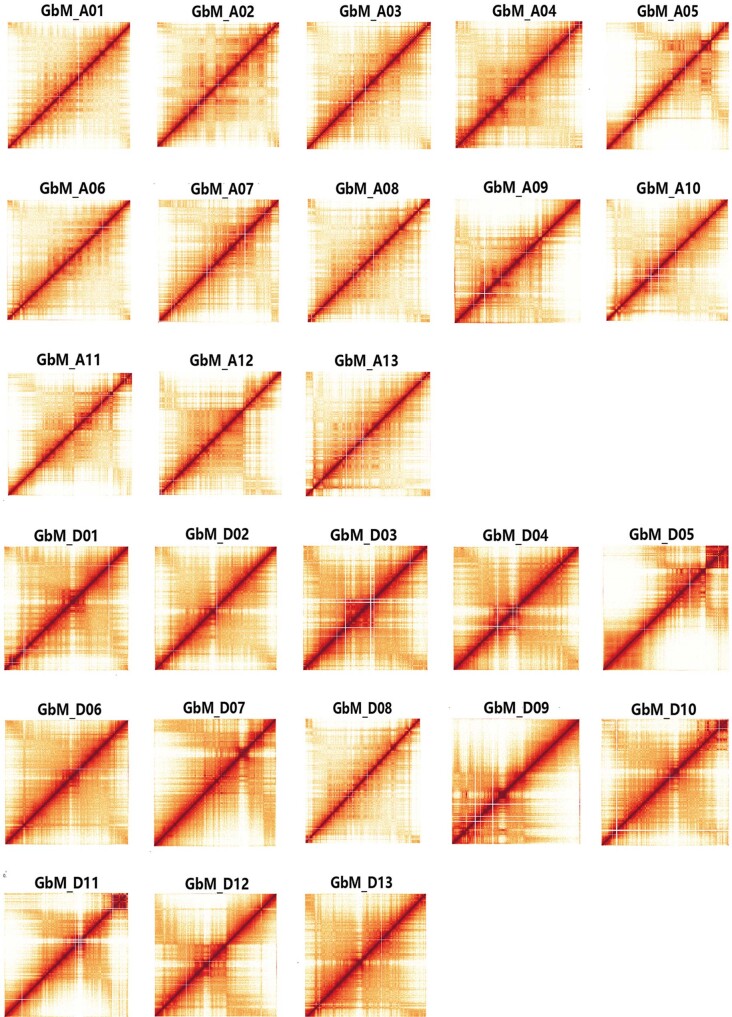
Chromatin interactions in each chromosome of *G. barbadense* Pima90. Each heatmap is shown at a resolution of 100 kb. The dark red dots show the high probability of interaction, and the light dots show the low probability of interaction.

**Extended Data Fig. 3:**
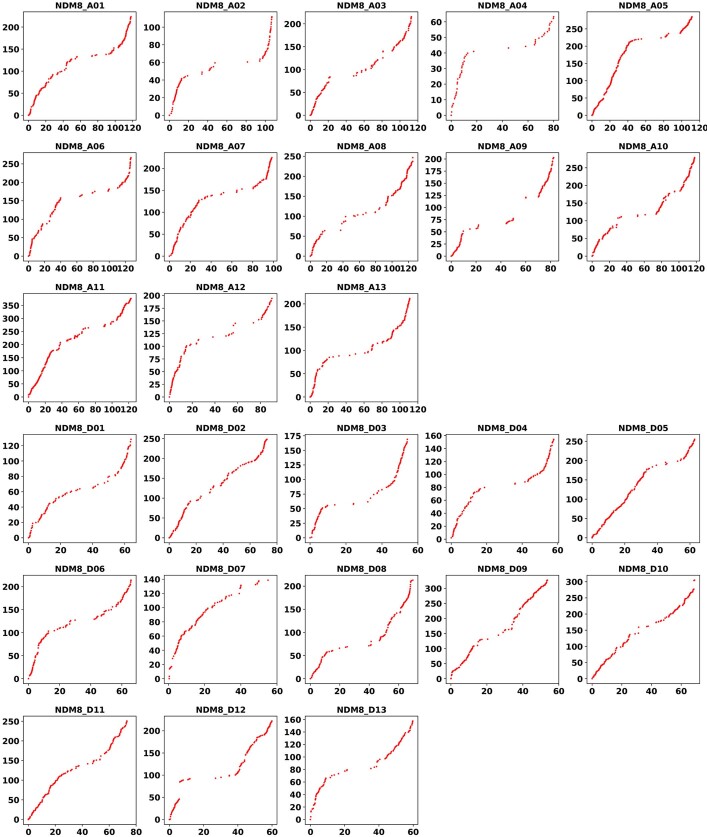
Comparison of Hi-C directed chromosome assembly with a published genetic map between *G. hirsutum* and *G. barbadense* for each chromosome in NDM8. The x-axes represent the physical positions of the sequences (Mb) and the y-axes represent the positions of the sequences on the genetic map (cM).

**Extended Data Fig. 4:**
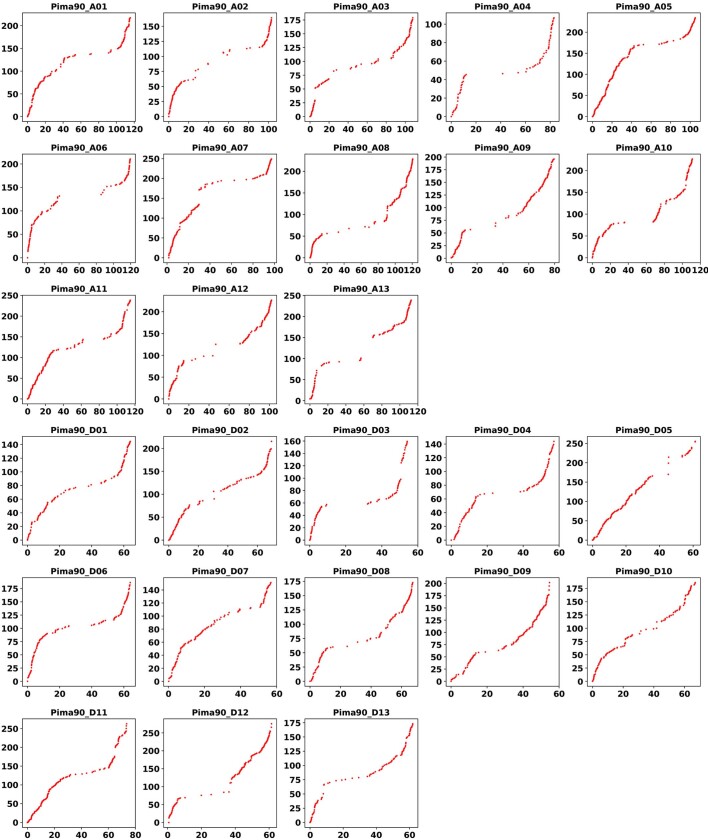
Comparison of Hi-C directed chromosome assembly with a published genetic map between *G. hirsutum* and *G. barbadense* for each chromosome in Pima90. The x-axes represent the physical positions of the sequences (Mb) and the y-axes represent the positions of the sequences on the genetic map (cM).

We assembled 2.29 Gb and 2.21 Gb of the NDM8 and Pima90 genomes, respectively (Table 1). To accomplish this, we obtained 205.18 Gb and 200.62 Gb long reads of NDM8 and Pima90 genomes, respectively, representing 180.38-fold coverage depth in total on the basis of single-molecule real-time (SMRT) sequencing (Supplementary Table 1). The initial assembly corrected by Illumina paired-end data (233.75-fold coverage in total) resulted in contigs with an N50 size of 15.28 Mb for NDM8 and 9.65 Mb for Pima90 (Supplementary Tables 2 and 3). Subsequently, these corrected contigs were connected to 754 superscaffolds for NDM8 and 909 for Pima90 using a total of 232.90-fold 10x Genomics linked-read data (Supplementary Tables 2 and 4). Finally, we constructed chromosome-scale scaffolds using more than 125-fold Hi-C interacting unique paired-end data from each cotton genome (Extended Data Figs. 1 and 2 and Supplementary Table 2). The final assemblies included 353 scaffolds for NDM8 and 309 for Pima90, resulting in contig and scaffold N50 values of 13.15 Mb and 107.67 Mb for NDM8 and 9.24 Mb and 102.45 Mb for Pima90 (Supplementary Table 5). A total of 99.57% and 99.75% of genomes were anchored onto pseudochromosomes in NDM8 and Pima90, respectively, and the very few gaps (0.003% in NDM8 and 0.06% in Pima90) indicated the contiguity of the sequences (Supplementary Table 6). High mapping ratios (99.16% in the two genomes) and low error assembly site ratios (1.87 × 10^−7^ in NDM8 and 2.95 × 10^−7^ in Pima90) indicated the accuracy of the genomes (Supplementary Tables 7 and 8). Besides, 96.1% and 95.9% of 1,440 embryophyta Benchmarking Universal Single-Copy Orthologs (BUSCOs) present in NDM8 and Pima90, respectively, showed the integrity of the genomes (Supplementary Table 9). We compared our two genomes to a published genetic map^17^, and a high consistency for each chromosome was validated for both genomes (Extended Data Figs. 3 and 4). Further, the accuracy and completeness of NDM8 assembly was confirmed by perfect alignment to 36 bacterial artificial chromosome sequences^4–6^ (Supplementary Table 10). Moreover, the centromeric regions of NDM8 and Pima90 were well collinear with those of the published genomes^5^ (Supplementary Tables 11 and 12). Comparing NDM8 with TM-1 (ref. ^4^) and ZM24 (ref. ^6^), and Pima90 with 3–79 (ref. ^4^) showed a high collinearity of more than 99.69% (Supplementary Fig. 1 and Supplementary Table 13). The higher long terminal repeat (LTR) assembly index (LAI) scores^18,19^ (14.2 in NDM8 and 12.1 in Pima90), as well as greater contig N50 sizes and fewer gaps in our two genomes (Supplementary Table 14) indicated that we had assembled high-quality *G.* *hirsutum* and *G.* *barbadense* genomes.

**Table 1:** Global summary of the final genome assemblies for NDM8 and Pima90

Genomic features	NDM8	Pima90
Assembled genome size (Mb)	2,291.77	2,210.14
A-subgenome (Mb)	1,438.06	1,381.46
D-subgenome (Mb)	843.88	823.21
Anchoring (%)	99.57	99.75
Number of contigs	1,030	1,160
Contig N50 (Mb)	13.15	9.24
Scaffold N50 (Mb)	107.67	102.45
Gap ratio (%)	0.003	0.06
GC content (%)	34.36	34.17
Repeat ratio (%)	62.10	61.85
Predicted PCG model number	80,124	79,613
Average gene length (bp)	2,931.27	2,894.70
Average coding sequence length per gene (bp)	1,088.90	1,094.24
Average exon number per gene	4.76	4.77
BUSCOs (%)	96.1	95.9

We identified 80,124 and 79,613 protein-coding gene (PCG) models in NDM8 and Pima90, respectively (Table 1 and Supplementary Tables 15 and 16), with 78,509 (98.61%) expressed PCG models in NDM8 and 78,980 (98.57%) in Pima90 on the basis of the transcriptome data from our laboratory and published data^4,5,20,21^ (Supplementary Data Files 1 and 2). Compared with the PCG models from the genomes of TM-1 (refs. ^4,5,20^), ZM24 (ref. ^6^), Hai7124 (ref. ^5^) and 3–79 (ref. ^4^), and the A genome^7,22^ and D genome^23^, 96.98% and 97.42% of homologous PCG models had a good match, with more than 80% identity of protein sequences in NDM8 and Pima90, respectively (Supplementary Table 17). We found 1,499 and 1,267 newly predicted PCG models (identity of protein sequences <20%) in NDM8 and Pima90, respectively. Of them, 96.5% in NDM8 and 92.5% in Pima90 could be transcribed in *G.* *hirsutum* and *G.* *barbadense*, respectively (Supplementary Tables 18 and 19). Further, we discovered that NDM8 and Pima90 had lost 1,324 and 2,318 genes when compared with TM-1 (ref. ^4^) and 3–79 (ref. ^4^), of which 635 and 1,605 had functional annotations, respectively (Supplementary Tables 20 and 21).

We analyzed the frequency of 1,499 *G.* *hirsutum* newly predicted gene models in 1,081 resequenced accessions and their expression in the closely related species *G.* *arboreum* Shixiya1 (ref. ^24^) and *G.* *barbadense* Pima90 and Hai7124. We found that 95.26% of the genes were harbored by at least 900 accessions (Supplementary Table 22), and 87.53% expressed in at least one variety and 100% in at least one tissue (Supplementary Table 23). Of 1,267 *G.* *barbadense* newly predicted genes, 90.53% were transcribed in at least one variety among Shixiya1 (ref. ^24^) and five *G.* *hirsutum* varieties and 92.66% in at least one tissue (Supplementary Table 24).

We predicted 1,263.36 Mb and 1,204.74 Mb LTRs, which are paramount in the evolution and domestication of crops^25,26^, and they covered 55.13% of NDM8 and 54.51% of Pima90 genomes (Supplementary Table 25). Of these, *Copia* was present to a much lesser extent than *Gypsy* in the NDM8 genome (17.82% versus 81.29%, *P* = 5.97 × 10^−27^, Mann–Whitney *U*-test), as was also the case in Pima90 (18.14% versus 81.07%, *P* = 2.26 × 10^−26^, Mann–Whitney *U*-test) (Supplementary Fig. 2 and Supplementary Table 26). We found that the number of genes with *Copia* and *Gypsy* insertions (14,900 and 14,628) was almost the same in the two genomes, and 96.69% and 95.05% of these genes were supported by transcriptome data, respectively (Supplementary Tables 26–28). The expressed gene number per *Copia* insertion was 1.84 × 10^−2^ and 4.68 times that per *Gypsy* insertion (3.92 × 10^−3^), showing that the *Copia* impact power might be greater than that of *Gypsy*. This was further evidenced by the fact that the gene number per *Copia* insertion to exonic and promoter regions was 9.48 × 10^−2^ and 3.73 times that per *Gypsy* insertion (2.54 × 10^−2^), which was also supported by the finding that *Copia* was markedly more active than *Gypsy* in the recent 0–1 MYA time frame^27^.

We further analyzed the effects of *Copia* and *Gypsy* insertion on the gene expression of tetraploid cultivated cottons. We focused on all homologous genes between *G.* *barbadense* and *G.* *hirsutum*, and found thousands of genes diversified in *Copia* and/or *Gypsy* insertion, with 6,306 genes only in *G.* *barbadense* and 5,268 only in *G.* *hirsutum*. Additionally, *G.* *barbadense* had more expressed genes (5,457) but at a lower percentage (86.54%) than *G.* *hirsutum* (4,841, 91.89%) during fiber development. Similar trends that 82.48% genes expressed in *G.* *barbadense* versus 87.81% in *G.* *hirsutum* under *Verticillium dahliae* (Vd) stress were found. The percentage of upregulated genes (26.50% for fiber and 22.55% for Vd) was lower than that of downregulated genes (40.02% for fiber and 47.63% for Vd) in *G.* *barbadense*, whereas the opposite was true in *G.* *hirsutum* (Supplementary Tables 29–31). These findings indicated that *Copia* and *Gypsy* played important roles in agronomic character diversification during the evolution of both cotton species.

### Genomic structural variations in Pima90 against NDM8

To potentially and effectively use the genomic variation of *G.* *barbadense* in modern *G.* *hirsutum* breeding programs, we aligned the Pima90 assembly onto the NDM8 genome and found high genomic diversification (Supplementary Fig. 3 and Supplementary Table 32). We discovered 78,126 gene models in Pima90 homologous to 78,238 in NDM8. For the nonhomologous gene models, 1,394 were in syntenic blocks and 93 in nonsyntenic blocks (Supplementary Table 33), with 62.81% such genes expressed in several tissues (Supplementary Table 34). In total, we detected 846,363 structural variations in Pima90, with 517,230 insertions and 317,638 deletions. The top three numbers of both insertion and deletion were found on the At12, At09 and Dt11 chromosomes (t in At or Dt indicates tetraploid). Insertions and deletions ≤10 bp occupied 94.34% of the total (Supplementary Table 35). The total number of insertions and deletions in At (418,107) was almost equal to that in Dt (416,761); however, the densities of insertions (312 per megabase) and deletions (194 per megabase) in Dt were evidently higher than those in At (188 per megabase and 114 per megabase, respectively) (*P* = 6.43 × 10^−13^ for insertions and *P* = 1.51 × 10^−13^ for deletions, Mann–Whitney *U*-test) (Supplementary Fig. 4).

**Extended Data Fig. 5:**
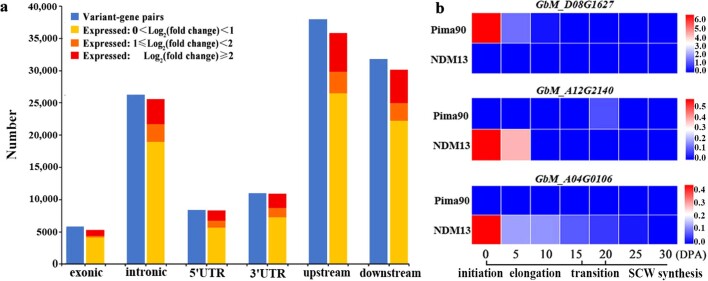
The number of differentially expressed genes in variant-gene pairs. **a**, The number of differentially expressed genes with the insertion and deletion in gene and/or regulatory regions. **b**, The expression of *GbM_D08G1627*, *GbM_A12G2140* and *GbM_A04G0106*.

We analyzed expression changes for the insertion and deletion variant-gene pairs between *G.* *barbadense* and *G.* *hirsutum*, reflecting structural variation effect on gene expression^10^. On the basis of our transcriptome data between *G.* *barbadense* and *G.* *hirsutum*, from different fiber developmental stages, tissues (root, stem and leaf) and inoculation time-points with Vd, we found that 31,296 variant-gene pairs (the variants in genes and/or ±1 kb flanking regulatory regions) showed significantly differential expression (log_2_ fold-change ≥1, *P* ≤ 0.05) (Extended Data Fig. 5 and Supplementary Table 36), indicating that the structural variations might, to some extent, affect gene expression. Three variant-gene pairs can be exampled. Two 1-bp insertions and a 1-bp deletion located in the introns of an EXPANSIN gene *GbM_D08G1627* whose homologous protein functioned in improving fiber length (FL) and micronaire value (M)^28^. This gene was expressed in *G.* *barbadense* only during the fiber elongation period. Insertions of 8-bp and 1-bp were located downstream in *GbbHLH* (*GbM_A12G2140*), as were four insertions and four deletions in the introns and downstream of *GbDIR* (*GbM_A04G0106*). Both genes are positive regulators involved in lignin biosynthesis; however, excessive lignin in the cell walls of cotton fibers restricts elongation and secondary cell wall (SCW) synthesis^29,30^. The null expression of *GbbHLH* and *GbDIR* might be related to better fiber quality (Extended Data Fig. 5).

**Extended Data Fig. 6:**
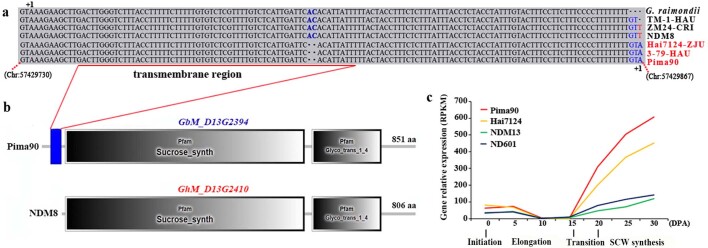
The structure of sucrose synthase (Sus) gene in Pima90 and NDM8, and expression analysis of different stages in cotton fiber development. **a**, Comparison of *Sus* gene sequences among ancestral diploid species and cultivated tetraploid cottons. **b**, The conservative structures of the *Sus* in Pima90 and NDM8, respectively. The blue shadow rectangle indicated transmembrane region within *GbM_D13G2394*. **c**, The transcriptome of *Sus* gene in cotton varieties with different fiber quality during fiber developmental stages. The *Sus* in Pima90 with super fiber quality showed higher expression level than that in NDM8 (good fiber quality) and ND601 (common fiber quality).

We found 5,815 variants in the exons of 5,256 genes, with 4,180 variants causing frameshift and 381 causing the gain or loss of a stop codon in Pima90 (Supplementary Table 37). A total of 3,178 variants were consistent with the transcripts from fiber, root, stem, leaf and Vd-infected tissues in *G.* *barbadense* and *G.* *hirsutum*. Among these genes, we discovered that *GbM_D13G2394*, encoding sucrose synthase (Sus), which plays a principal role in cotton fiber elongation and/or SCW synthesis^31,32^, contained a transmembrane domain with a 2-bp deletion in Pima90; the *GbSus* expression was distinctly higher during fiber elongation and SCW synthesis in *G.* *barbadense* (Extended Data Fig. 6). This indicated that the new isoform of *GbSus* may play a crucial role in *G.* *barbadense* fiber length and strength. This 2-bp deletion was also identified in 3–79, Hai7124 and two *G.* *barbadense* introgression lines NDM373-9 and Luyuan343 (ref. ^33^) with good fiber quality.

We identified 9,515 inversions with an average of 21.85 kb distributed nonrandomly across Pima90 chromosomes (Supplementary Fig. 5 and Supplementary Table 38). Of those, 6,685 and 2,830 inversions were located in At and Dt, respectively, with higher density in At (4.84 × 10^−3^ per kilobase) than in Dt (2.71 × 10^−3^ per kilobase) (*P* = 6.44 × 10^−9^, Mann–Whitney *U*-test). The top three numbers of inversion were found on At06, At08 and At12, which differed from the case in 3–79 (ref. ^4^). The largest inversion (585.02 kb) was located on At05, whereas the largest inversion in 3–79 (328.2 kb) was seen on Dt12. We discovered that 2,024 inversions overlapped with the exons of genes, which might lead to gene function changes (Supplementary Table 39). Additionally, we detected 1,980 translocations, of which 74.09% were interchromosomal (Supplementary Table 40).

To illustrate the potential use of *G.* *barbadense* germplasm in *G.* *hirsutum* breeding, we resequenced (30-fold) a *G.* *hirsutum* new line, NDM373-9, developed through backcross with the donor parent Pima90 and exhibited better Verticillium wilt (VW) resistance and fiber properties than its receptor parent *G.* *hirsutum* CCRI8 (Supplementary Fig. 6 and Supplementary Table 41). We found that NDM373-9 contained 171 exonic structural variations transferred from Pima90, and 34 and 12 genes with such structural variations were related to disease resistance and fiber development, respectively, as reported in previous studies (Supplementary Table 42).

### Genomic structural variations in *G.hirsutum* NDM8

**Fig. 1:**
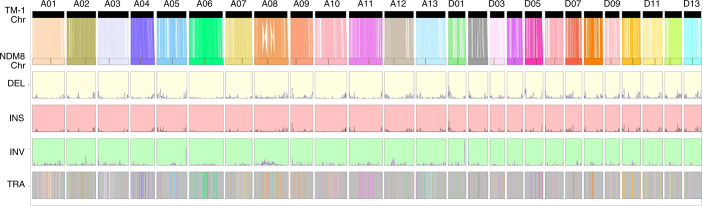
Genomic landscape of NDM8 and TM-1_HAU genomes. The vertical lines indicate the synteny between two genomes. Chr, Length of chromosome (Mb); DEL, density distribution of deletions; INS, density distribution of insertions; INV, density distribution of inversions; TRA, translocations between NDM8 and TM-1. The sliding windows are nonoverlapped with a 500-kb length.

The high-quality genome of NDM8 allowed us to understand the genomic changes of modern *G.* *hirsutum* through comparison with TM-1 (ref. ^4^), the two cultivars being released more than half a century apart (Supplementary Fig. 7). We identified 76,568 structural variations in NDM8 (Fig. 1 and Supplementary Table 43), including 27,708 insertions, 47,221 deletions, 808 inversions and 831 translocations. Further, we detected 28,626 consistent structural variations supported by the accessions ranging from 10 to 1,081 in the resequencing population (Supplementary Table 44).

**Fig. 2:**
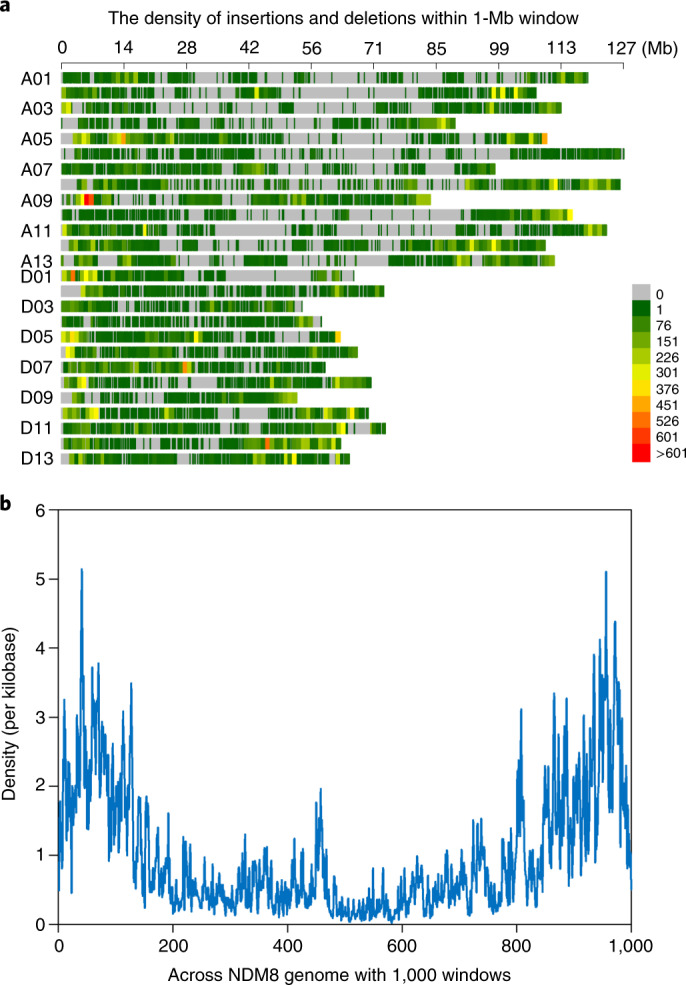
Density distribution of insertions and deletions in NDM8 genome. **a**, The density of insertions and deletions in a 1-Mb window of chromosomes. **b**, The density of insertions and deletions across NDM8 genome with 1,000 windows.

**Extended Data Fig. 7:**
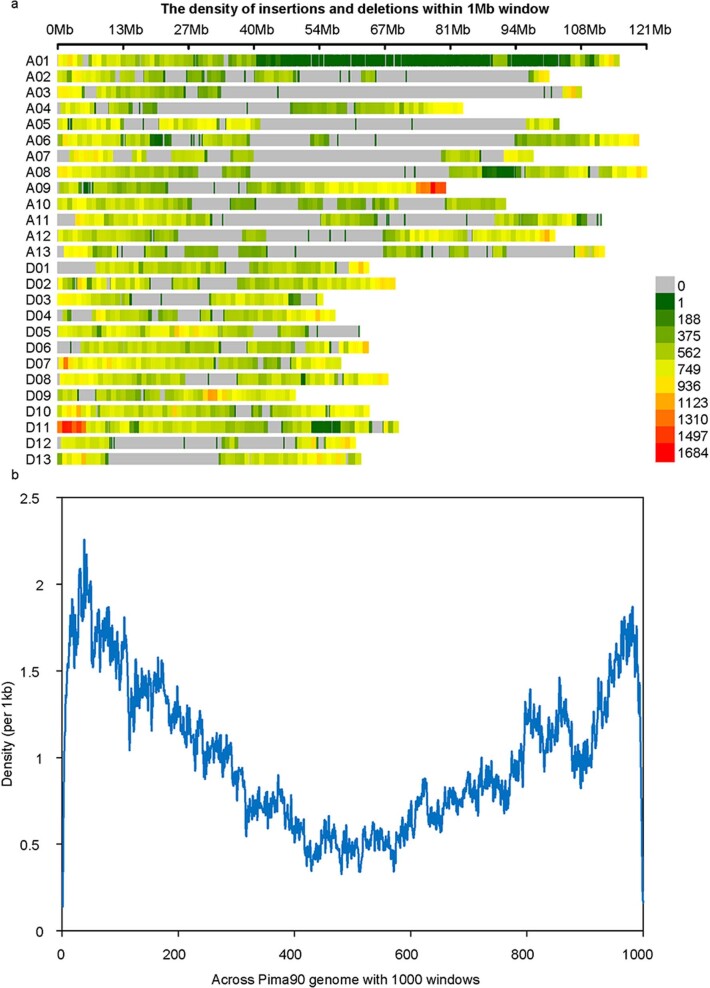
Density distribution of insertions and deletions in Pima90. **a**, The density of insertions and deletions within 1 Mb window of chromosomes. **b**, The density of insertions and deletions across Pima90 genome with 1,000 windows.

We found that the numbers of insertions (13,985) and deletions (23,677) in At were roughly equal to those in Dt (12,705 insertions and 21,076 deletions); however, the densities of insertions and deletions were apparently higher in Dt (*P* = 1.28 × 10^−3^ for insertions and *P* = 3.18 × 10^−4^ for deletions, Mann–Whitney *U*-test) (Supplementary Fig. 8), which was also observed in the comparison of Pima90 against NDM8. We further analyzed the density of insertions and deletions across each chromosome, and observed the strongest bias within 20% of the windows near the telomeres, with a 3.71-fold (*P* < 10^−6^, permutations) increase over that in the other regions (Fig. 2). This was much higher than that of Pima90, with a 1.89-fold increase (Extended Data Fig. 7).

**Extended Data Fig. 8:**
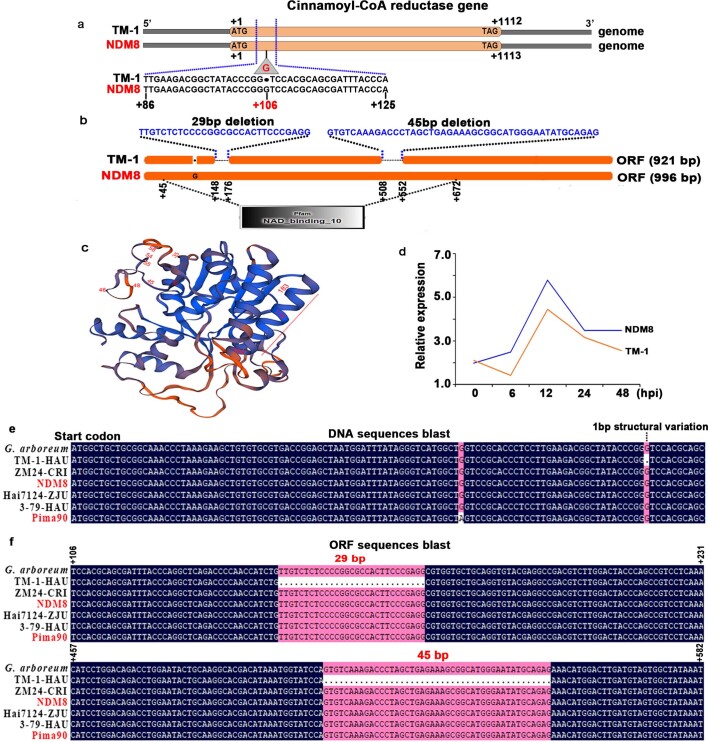
The structural variation of *CCR* gene (*GhM_A02G1731* versus *Ghir_A02G014590*). **a**, The location of structural variation in the genome of NDM8 against TM-1. **b**, The structural variation led to the difference in the open reading frame (ORF) between NDM8 and TM-1, and the conservative structure domain (NAD_binding_10) of *CCR* in NDM8. **c**, Three-dimensional structure of CCR (GhM_A02G1731) was obtained by homologous modeling. The second deletion (508–552) in TM-1 influenced the formation of *CCR* structure within NAD-binding domain that was indicated by red dotted line. **d**, Expression of *CCR* in resistant (NDM8) and susceptible (TM-1) cotton varieties under *V. dahliae* stress through qRT–PCR. *Ghhistone3b* was used as an internal control. **e**, Comparison of *CCR* genomic sequences among ancestral diploid species and cultivated tetraploid cottons. **f**, Comparison of *CCR* partial coding sequences among ancestral diploid species and cultivated tetraploid cottons.

Furthermore, we found 603 insertions and deletions in the exons of 526 genes in NDM8 (Supplementary Table 45). Among these genes, 189 were homologous, 76 were nonhomologous and 261 were not annotated genes in the corresponding positions of TM-1, which might potentially indicate gene function changes. For example, of the 189 genes, *GhM_A02G1731* in NDM8 is homologous to the rice cinnamoyl-CoA reductase (CCR) gene that plays a role in fungal disease resistance by controlling lignin synthesis^34,35^. However, the gene in VW-susceptible TM-1 contained a 1-bp deletion in splicing site, resulting in two deletions (29 bp and 45 bp) and a truncated protein with an impaired NAD-binding domain and a lower expression level under Vd stress than that in VW-resistant NDM8 (Extended Data Fig. 8).

Of 808 inversions, the largest inversion of 1.77 Mb was located in At08, and 257 overlapped with gene models (Supplementary Tables 46 and 47). The number of inversions in At was 2.62 times that in Dt, which did not match with the fact that the genome of At was 1.70 times that of Dt, showing significantly higher density in At (*P* = 2.60 × 10^−5^, Mann–Whitney *U-*test) (Supplementary Fig. 9), in contrast to the case that insertions and deletions were situated mainly in Dt in both Pima90 and NDM8. We detected that 57.52% of 831 translocations were interchromosomal (Supplementary Table 48).

Furthermore, we found 4,984 ordered genes without any structural variation (100% identity) (Supplementary Table 49) in 159,960 identical ordered synteny blocks (no gap, no mismatch and each ≥1 kb) in NDM8 (Supplementary Fig. 10), indicating that these genes might be important in maintaining fundamental biological characteristics. In addition, we made a comparison between NDM8 and ZM24 (ref. ^6^), and obtained 1,393 insertions, 9,113 deletions, 243 inversions and 146 translocations (Supplementary Table 50). For the length of inversion and translocation, we found NDM8 versus ZM24 < ZM24 versus TM-1 < NDM8 versus TM-1 (Supplementary Table 51), indicating that the closer the breeding-year of two varieties were, the fewer the variations.

We analyzed the structural variations in 100 early varieties (released before 1970 and developed mainly through pedigree selection) and 100 modern varieties (released after 1990 and developed mainly through cross breeding) that were significantly improved in economic traits (Supplementary Table 52). We found that the modern varieties acquired 1,128 structural variations (in at least 51% of the varieties) compared with the early varieties during breeding (Supplementary Table 53). We found 555 and 573 acquired structural variations in At and Dt, respectively, whereas a higher density was observed in Dt (6.79 × 10^−4^ per kilobase) than in At (3.86 × 10^−4^ per kilobase) (*P* = 7.81 × 10^−5^, Mann–Whitney *U-*test), implying that Dt underwent stronger selection during modern breeding.

### Structural variations associated with agronomic traits in *G.hirsutum*

We explored structural variations by resequencing 1,081 *G.* *hirsutum* accessions (average 10.65-fold) referring to the NDM8 genome (Supplementary Table 54). On the basis of strict screening, we obtained 304,630 structural variations, including 141,145 insertions, 156,234 deletions, 39 inversions, 6,384 translocations and 828 duplications (Supplementary Table 55); 76.94% were located in intergenic regions, and the variation percentage was lower in coding sequences than in intronic regions (Supplementary Table 56). The structural variations, together with 2,970,970 SNPs and genetic kinship of all the accessions (Supplementary Fig. 11 and Supplementary Tables 57 and 58), provided broad molecular basis for cotton improvement.

**Extended Data Fig. 9:**
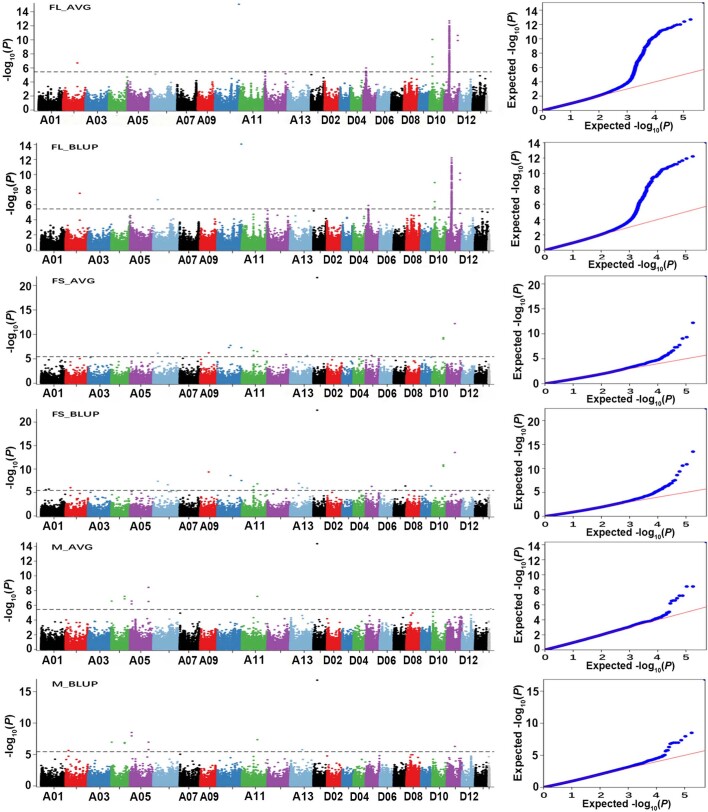
GWAS of fiber quality related traits based on accessions and structural variations. Manhattan plots and Quantile-Quantile plots using mean (AVG) and BLUP values of all environments. The genome-wide significant -log_10_(*P*) = 5.44 is indicated by the gray dotted line. FL, fiber length; FS, fiber strength; M, micronaire value. The statistical analysis was performed with two-tailed Wald test.

**Extended Data Fig. 10:**
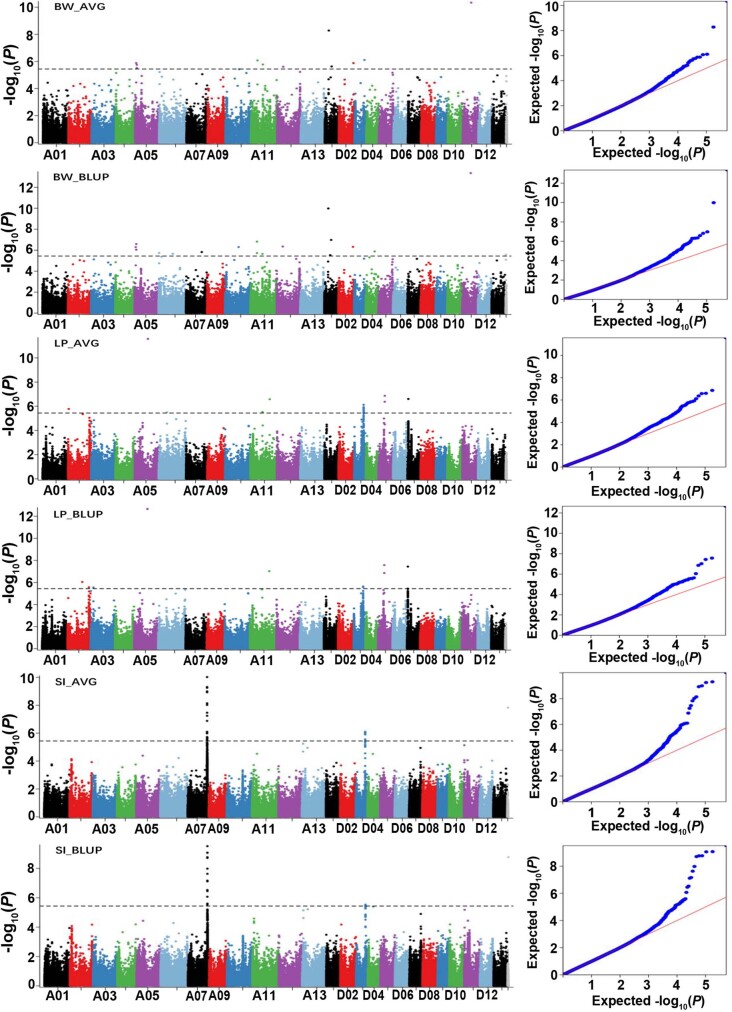
GWAS of yield related traits based on accessions and structural variations. Manhattan plots and Quantile-Quantile plots using mean (AVG) and BLUP values of all environments. The genome-wide significant -log_10_(*P*) = 5.44 is indicated by the gray dotted line. BW, boll weight; LP, lint percentage; SI, seed index. The statistical analysis was performed with two-tailed Wald test.

So far, the genetic effects of structural variations underlying agronomically important traits remain elusive in cotton. Thus, we conducted a genome-wide association study (GWAS) for principal fiber quality and yield traits and VW resistance. The best linear unbiased prediction (BLUP) values and means for each of six traits, including FL, fiber strength (FS), M, boll weight (BW), lint percentage (LP) and seed index (SI) were calculated on the basis of phenotypic data from several environments representing years and locations (14 environments for the core collection of 419 accessions^8^, eight environments for the 662 expanded accessions^36^ and one environment for all 1,041 accessions in 2019). For VW resistance, the disease index (DI) of 401 accessions was determined using the high-pathogenicity Vd strain LX2-1 (ref. ^37^) in a growth chamber with four independent experiments. We identified 446 structural variations significantly associated with the seven traits, of which 346 with fiber quality, 97 with yield and 3 with VW resistance (Extended Data Figs. 9 and 10 and Supplementary Data File 3). We focused on 193 structural variations simultaneously detected by both BLUP and average values (hereafter the same), and found 160 and 33 structural variations for fiber quality and yield traits, respectively. There are 29 variations in regulatory regions and 19 in genes that need to be the focus of functional analyses because they can directly alter the functionality of transcriptional regulatory elements and genes. The structural variations for fiber quality traits (FL, FS, M) were situated mainly in Dt (139 versus 21 in At), whereas those for yield traits (BW, LP, SI) were situated mainly in At (22 versus 11 in Dt).

**Fig. 3:**
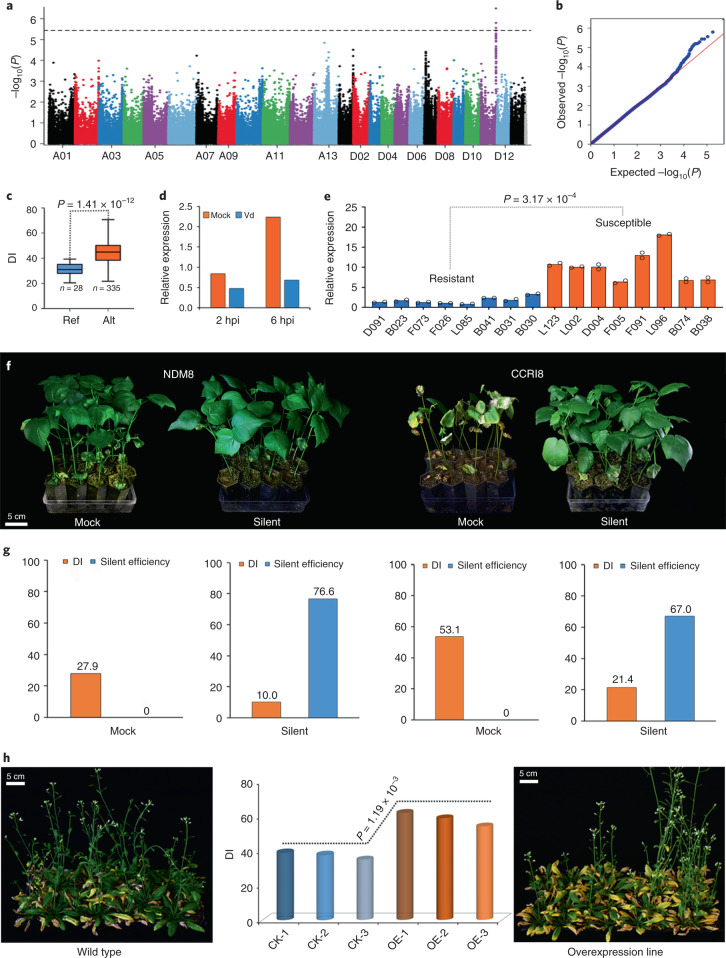
Identification of the causal gene *GhNCS* related to VW resistance on chromosome Dt11. **a**, Manhattan plot. Dashed line represents the significance threshold (−log_10_(*P*) = 5.44). We performed statistical analysis with a two-tailed Wald test. **b**, Quantile-quantile plot. **c**, Boxplot for DI on the basis of structural variation (D11: 69329075). In the box plots, the center line denotes the median, box limits are the upper and lower quartiles and whiskers mark the range of the data; *n* indicates the number of accessions with the same genotype. The difference significance was analyzed by two-tailed *t-*test. **d**, Expression level of *GhNCS* in resistant variety ND601 inoculated with Vd LX2-1. **e**, qRT–PCR analysis of *GhNCS* in eight resistant and eight susceptible varieties under Vd stress. *Ghhistone3b* was used as an internal control. The data were analyzed from the total of 16 varieties and expressed as the mean from two experiments. The difference significance was analyzed by two-tailed *t-*test. **f**, Silencing of *GhNCS* in tolerant variety NDM8 and susceptible variety CCRI8 led to obviously increased resistance compared with the mock. Scale bar, 5 cm. **g**, For each independent virus-induced gene silencing experiment, 35 cotton seedlings with higher silent efficiency were used for VW disease resistance detection. **h**, *GhNCS* overexpressed in *Arabidopsis* made transgenic plants highly susceptible compared with the wild type. Scale bar, 5 cm.

For FL, which can markedly increase the economic value of end-use yarns in the textile industry, we detected the highest association peak in Dt11, where a 370-kb region (24.55–24.93 Mb) harbored 125 structural variations. Among these loci, as in NDM8, 69 and 56 increased FL significantly by 0.71–0.99 mm and by 1.00–1.19 mm, respectively (Supplementary Table 59), increasing FL from 27-mm or 28-mm grade to 29-mm grade. For the important lint yield trait LP, two structural variations in Dt03 increased LP significantly from 37.49% to 39.69% and from 37.47% to 40.00%. For VW resistance, a peak in Dt11 (69.00–69.33 Mb) with three structural variations caused a DI decline of more than 13.6 in the genotype, the same as the resistant NDM8, shifting the disease reaction from susceptible (DI = 44.5–45.2) to tolerant (DI = 30.9–31.1) (Fig. 3a–c).

We identified 907 candidate genes for fiber quality and yield traits and 60 for VW resistance on the basis of a linkage disequilibrium decay value of 325 kb (Supplementary Fig. 12). We found 84.23% genes expressed at the fiber developmental stages of *G.* *hirsutum*, of which 305 had structural variations in genes and regulatory regions (Supplementary Data File 3), implying that these genes might potentially influence fiber quality and yield. Moreover, we found that four deletions in the 5′ untranslated region (UTR), intronic and 3′ UTR of *GhM_D11G2206* were significantly associated with FL. This gene was the same as the validated *GhFL2* in our previous study^8^.

To validate the reliability of GWAS results for significant hits, we chose the gene *GhM_D11G3743* associated with two structural variations in Dt11. This gene encodes (*S*)-norcoclaurine synthase, designated as *GhNCS*, and is a member of the pathogenesis-related 10/Bet v1 protein family^38^ whose function in cotton disease resistance is unclear. qRT–PCR assays showed that *GhNCS* expression was downregulated under Vd stress compared with mock and significantly lower amounts in eight resistant varieties (reference genotype) than in eight susceptible varieties (alternative genotype) (Fig. 3d,e and Supplementary Table 60). Silencing *GhNCS* in cotton resulted in resistance enhancement in both susceptible and resistant varieties, making the highly susceptible variety CCRI8 (DI = 53.1) tolerant (DI = 21.4) and the tolerant variety NDM8 (DI = 27.9) resistant (DI = 10.0) (Fig. 3f,g). Nevertheless, overexpression of *GhNCS* in *Arabidopsis* made the transgenic plants highly susceptible (DI = 58.1) compared with the wild type (DI = 38.1) (Fig. 3h). These results indicate that *GhNCS* is a plausible causal gene controlling VW resistance and that the associated structural variations are reliable.

## Discussion

In the present work, we completed two new high-quality assemblies of modern *G.* *hirsutum* cv. NDM8 and *G.* *barbadense* acc. Pima90, and detected many interspecific and intraspecific genomic variations. More and larger inversions occurred in the A-subgenome of *G.* *hirsutum*, which was similar to the recent reports^6,20,39^; however, the D-subgenome acquired more insertions and deletions than the A-subgenome during modern breeding. The density of insertions and deletions across each chromosome showed the strongest bias near the telomeres, similar to what has been reported in the human genome^10^. These will enhance the genomic resources for cotton improvement and provide insight into species formation and variety development.

There are several reports about the genomic diversity of *Gossypium* allopolyploid species on the basis of sequencing *G.* *hirsutum* TM-1, ZM24, *G. barbadense* Hai7124, 3–79, *G.* *tomentosum*, *G.* *mustelinum* and *G.* *darwinii*^4–6,39,40^ and resequencing large-scale accessions^8,41^. On the basis of the sum of the gene number in each gene family counting by the priority in 3–79 > TM-1_HAU > Hai7124 > TM-1_ZJU > ZM24 > TM-1_CRI tetraploid cottons, we found that 15,973 genes might actually belong to duplicates and/or alleles of some genes, and 80,992 were nonredundant in the six genomes (Supplementary Table 61), which provides new information for plant genome researchers.

We found that a 2-bp deletion in *GbSus* in the D-subgenome of Pima90 (also existed in 3–79 and Hai7124) diverged from species formation because the deleted AC bases could be detected in the D-subgenome of NDM8, TM-1 and ZM24 and traced in the ancestral diploid species *G.* *ramondii* (Extended Data Fig. 6). Similarly, a 1-bp insertion in *CCR* in the A-subgenome of NDM8 could be found in Pima90, 3–79, Hai7124 and ZM24 and traced in the ancestral diploid species *G.* *arboreum* Shixiya1 (Extended Data Fig. 8). We inferred that NDM8 regained the insertion from its pedigree ancestral varieties, excluding TM-1 and its selections, during artificial recombination in breeding.

## Methods

### Plant material and resequencing

*G.* *hirsutum* cv. NDM8 and *G. barbadense* acc. Pima90 (self-pollinated for more than ten generations) were selected for genome sequencing because of their important roles in cotton genetic research and breeding. NDM8 was released in 2006, with high yield, good fiber properties and resistance to Fusarium wilt and VW. Pima90 is selected from Pima cotton. A total of 1,081 *G.* *hirsutum* accessions from China and other countries were used for resequencing according to our previous description^8^ (Supplementary Table 55). After germination, five full seeds of each accession were planted in pots with vermiculite and cultured at 27 °C in a growth chamber. After two cotyledons spread, the cotyledons of a single seedling were harvested and frozen immediately in liquid nitrogen for the extraction of genomic DNA.

### Genomic DNA for PacBio

Total genomic DNA from two cottons, NDM8 and Pima90, was extracted for sequencing using the CTAB method. To construct sequencing libraries, genomic DNA was fragmented by g-TUBE, centrifuged at 2,000 r.p.m. for 2 min, and treated with end-repair, adapter ligation and exonuclease digestion as recommended by Pacific Biosciences. DNA fragments at 10–50 kb were selected by Blue Pippin electrophoresis (Sage Sciences). DNA libraries were sequenced on the PacBio Sequel platform (Pacific Biosciences) with Sequel Sequencing chemistry v.3.0. A total of 21 SMRT cells were sequenced for NDM8 producing 205.41 Gb of polymerase reads and 27 cells for Pima90 producing 200.82 Gb of raw data. For the PacBio data, subreads were filtered with the default parameters, and the N50 length of long subreads reached 19.84 kb and 18.82 kb in NDM8 and Pima90, respectively.

### Illumina paired-end sequencing

Genomic DNA of each accession was extracted (1.5 μg per sample) and used as input material for DNA sample preparation. Sequencing libraries were generated using a TruSeq Nano DNA HT Sample Preparation Kit (Illumina) following the manufacturer’s instructions, and index codes were added to attribute sequences to each sample. Briefly, the DNA samples were fragmented by sonication to short inserts (350 bp), and the DNA fragments were then end-polished, A-tailed and ligated with the full-length adapters for Illumina sequencing with further PCR amplification. Finally, PCR products were purified (AMPure XP), and the libraries were analyzed for size distribution using an Agilent 2100 Bioanalyzer and quantified using real-time PCR.

### 10x Genomics library construction, sequencing and extension scaffold

The GemCode Instrument from 10x Genomics was used for DNA sample preparation, indexing and barcoding. Around 1 ng of input DNA with a 50-kb length was used for the GEM reactions during PCR, and 16-bp barcodes were introduced into droplets. Then, the droplets were fractured following purification of the intermediate DNA library. Next, we sheared DNA into 500-bp fragments for constructing libraries, which were finally sequenced on NovaSeq.

### Hi-C library construction and sequencing

We constructed Hi-C libraries from cotton leaves of NDM8 and Pima90. The leaves were fixed with formaldehyde and lysed. After that, we digested the cross-linked DNA with *Hin*dIII. Sticky ends were biotinylated and proximity-ligated to form chimeric junctions. They were then enriched and physically sheared into fragments of 300–500 bp. The chimeric fragments representing the original cross-linked long-distance physical interactions were processed into paired-end sequencing libraries. Finally, 150-bp paired-end sequences were produced on the Illumina platform^42^.

### Sequence quality checking and filtering

We used strict filters to avoid reads with artificial bias for Illumina paired-end sequences, 10x Genomics linked reads and Hi-C data. First, low-quality paired reads (reads with ≥10% unidentified nucleotides (*N*); >10 nt aligned to the adapter, allowing ≤10% mismatches; >50% bases having phred quality <5 and putative PCR duplicates generated in the library construction process), which resulted mainly from base-calling duplicates and adapter contamination, were removed. Consequently, we obtained 32.24 Tb of high-quality data for collection, extension, chromosome-scale scaffolds and large-scale population analysis.

### Hi-C reads mapping, filtering and generation of contact matrices

Initial Hi-C data analyses including read mapping, filtering and bias correction were conducted by Hiclib (https://github.com/mirnylab/hiclib-legacy). High-quality paired-end reads were mapped to the two genomes by Bowtie2 (ref. ^43^) (with the ‘very-sensitive’ option) through iterative mapping. Mapped reads were filtered using Hiclib^44^ with default parameters, discarding the invalid self-ligated and unligated fragments and PCR artifacts. Valid Hi-C read pairs harbored more intrachromosomal (cis) interactions than interchromosomal (trans) interactions. Normalized interaction matrices were generated at four resolutions from low to high: 1 Mb, 500 kb, 100 kb and 40 kb.

### Genome assembly

First, the package ‘daligner’ of the FALCON assembler^45^ was used to self-correct PacBio long reads using the PacBio short reads less than 5,000 bp. Then, contigs of the two cottons were assembled using the package FALCON assembler on the basis of the error-corrected reads. The overlapped read pairs were used to construct a directed string graph following Myers’ algorithm. Contigs were constructed by finding the paths from the string graph. The preceding assemblies were polished by the consensus–calling algorithm Quiver^46^. We mapped Illumina paired-end reads to the contig assemblies and corrected them using the Pilon pipeline^47^. The corrected contigs were further connected to generate superscaffolds by 10x Genomics linked-read data using fragScaff software^48^. Linkage information of superscaffolds was obtained by aligning high-quality Hi-C data to the preceding assemblies using Bowtie2 software. Chromosome-scale scaffolds were anchored by linkage information, restriction enzyme site, and string graph formulation with the package LACHESIS^49^. Hi-C data were mapped to chromosome-scale scaffolds to assess the quality of assemblies using HiC-Pro software^50^ (v.2.10.0). The placement and orientation errors exhibiting obvious discrete chromatin interaction patterns were adjusted manually.

### Assessment of genome assembly quality

To validate the single-base accuracy of the genome assemblies, we realigned the high-quality 350-bp paired-end reads to the assemblies with BWA software^51^. More than 99.67% of the genome having a coverage depth ≥10 indicated an extremely high sequencing depth over the whole genome. We conducted variant calling with SAMtools^52^ and obtained homozygous SNP (that is, error assembly site). We used BUSCO analysis^53^ to assess genome completeness by searching against the embryophyta BUSCO (v.3.0).

### Genome repeat annotation

The repetitive sequences in the cotton genome were identified by a combination of homology searching and ab initio prediction. For homology-based prediction, we used RepeatMasker^54^ and RepeatProteinMask to search against Repbase. For ab initio prediction, we used Tandem Repeats Finder^55^, LTR FINDER^56^, PILER^57^ and RepeatScout^58^ with default parameters. The code used for the genome annotations of repetitive elements is deposited in the Zenodo DOI-minting repository^59^.

### Structural annotation of genes

Gene prediction was conducted through a combination of homology- and ab initio–based methods and by incorporating evidence from transcriptions. Proteins of plants, including *Gossypium hirsutum* (http://cotton.hzau.edu.cn/EN/download.php, http://ibi.zju.edu.cn/cotton/), *Gossypium barbadense* (http://cotton.hzau.edu.cn/EN/download.php, http://ibi.zju.edu.cn/cotton/), *Gossypium raimondii* (https://phytozome.jgi.doe.gov/pz/portal.html#!bulk?org=Org_Graimondii), *Gossypium arboreum* (ftp://bioinfo.ayit.edu.cn/downloads/), *Theobroma cacao* (GCF_000208745.1), *Oryza sativa* (R498, IGDBV2), *Glycine max* (GWHAAEV00000000), *Populus trichocarpa* (GCF_000002775.4) and *Arabidopsis thaliana* (GCA_000001735.1) were used as queries to search against two cotton genomes using TBLASTN^60^ with an E-value cutoff of 1 × 10^−7^. The BLAST hits were conjoined by Solar software^61^. Then, we removed conjoined query hits with <25% coverage and merged two hits with >50% overlap in length. Subsequently, GeneWise^62^ was used to predict the exact gene structure of the corresponding genomic region on each conjoined hit. Homology predictions were denoted as ‘Homology-set’.

For transcription evidence, RNA-seq data of the four cotton tissues root, stem, leaf, fiber and public data from nine tissues^20^ were used. Illumina RNA-seq data were assembled by Trinity^63^, and full-length nonchimeric transcripts were obtained using IsoSeq3 pipeline (https://anaconda.org/bioconda/isoseq3) on the basis of PacBio sequences. Subsequently, these transcripts were aligned against two cotton genomes by the Program to Assemble Spliced Alignment (PASA)^64^ with default parameters. Valid transcript alignments were clustered on the basis of genome mapping location and assembled into gene structures. Gene models created by PASA were denoted as PASA Trinity set (PASA-T-set). In addition, Illumina RNA-seq reads were mapped to the genome using Tophat^65^ to identify putative exonic regions and splicing junctions, and then Cufflinks^66^ was used to assemble the mapped reads into gene models (Cufflinks-set).

We performed ab initio prediction for coding regions in the repeat-masked genome using Augustus^67^, GeneID^68^, GenScan^69^, GlimmerHMM^70^ and SNAP^71^. Specifically, GeneID and GenScan with the self-trained model parameters (*A.* *thaliana*) were used to predict two masked cotton genomes; Augustus, SNAP and GlimmerHMM were trained by PASA-H-set gene models; Augustus, SNAP and GlimmerHMM were used to predict two masked cotton genomes.

Gene models generated from all the methods were integrated by EvidenceModeler^72^. Weights for each type of evidence were set as follows: PASA-T-set > Homology-set > Cufflinks-set > Augustus > GeneID = SNAP = GlimmerHMM = GenScan. A weighted and nonredundant gene set were further revised by PASA2 to generate untranslated regions and alternative splicing variation information. The code used for the genome annotations of gene structures is deposited in the Zenodo DOI-minting repository^59^.

### Functional annotation of protein-coding genes

Gene functions of PCGs were annotated by searching for functional motifs and domains of genes and the possible biological processes in the databases SwissProt^73^, Pfam^74^, NR database (from National Center for Biotechnology Information (NCBI)), Gene Ontology^75^ and Kyoto Encyclopedia of Genes and Genomes^76^.

### Estimating the theoretical gene number of tetraploid cotton genome

We carried out gene orthologous cluster analysis of tetraploid cottons using the published cotton PCG models from the genomes of 3–79_HAU, TM-1_HAU, Hai7124_ZJU, TM-1_ZJU, ZM24_CRI and TM-1_CRI. Specifically, for genes with alternative splicing sites, we chose the longest translation to represent each gene and filtered genes with fewer than 50 amino acids. To build a graph of PCGs, all-against-all BLASTP was used to determine similarities between all genes in the six cottons with an E-value of 1 × 10^−7^. Subsequently, we conjoined fragmental alignments to cluster gene pairs by the OrthoMCL^77^ method with the parameter ‘-inflation 1.5’. Finally, we obtained 47,147 gene clusters. The largest theoretical gene resource is the sum of the largest number of genes in each gene family counting by the priority (3–79_HAU > TM-1_HAU > Hai7124_ZJU > TM-1_ZJU > ZM24_CRI > TM-1_CRI). Next, to filter duplicates and/or alleles of some genes between/within six tetraploid cottons, we extracted alignment pairs from any pair of genomes and restricted a maximum of five hits per protein sequence to serve as input for the MCScanX algorithm^78^ that was used to detect high-confidence collinear blocks of coding genes and identify orthologous gene pairs. Finally, we filtered 15,973 genes that might actually belong to duplicates and/or alleles of some genes.

### Synteny gene identification

We identified synteny blocks through genome alignment applying the MUMmer program^79^ (v.3.2) with the command ‘nucmer --mum --maxgap=500 --mincluster=1000’. Meanwhile, protein sequences were compared for identifying homologous genes by using all-by-all BLASTP^60^ (v.2.2.26; by E-value ≤1 × 10^−7^ and identity ≥20%). Subsequently, we identified the homologous genes in one-to-one genomic synteny blocks through intersection using BEDTools^80^ (v.2.27). Finally, we defined those homologous gene patterns to be ordered genes.

### Genomic variation detection

To compare two genomes, we used smartie-sv software^81^ to detect insertions and deletions. To filter out spurious insertions and deletions, we separately aligned the reads onto two genomes using BWA^51^, and calculated the read coverage for each candidate variant. Then, different criteria were used to validate the candidates ≤50 bp and those >50 bp. Some candidates (≤50 bp) were supported by more than three gapped aligned reads and their predicted breakpoints and/or genotypes were perfectly consistent with the aligned reads. The other candidates (>50 bp) should have significant differences in S/P ratio (that is, the number of aligned single-end reads versus the number of aligned paired-end reads) between two genomes (*P* < 0.05, Fisher’s exact test) and were more than three times the s.d. of the insert size in length. We detected inversion and translocation on the basis of the reverse-pattern and nonsequential-pattern synteny of the two genomes, respectively.

For population genomic variations, we separately aligned the individual sequence onto the NDM8 genome using BWA and Sentieon softwares^82^ to detect SNPs (MAF ≥ 0.05, missing ratio ≤0.2, depth ≥3) and small structural variations including insertions and deletions ≤250 bp (MAF ≥ 0.05, missing ratio ≤0.2, depth ≥3), respectively. Subsequently, we identified potential large structural variations using an SVMerge pipeline^83^ by integrating calls from the packages LUMPY^84^ and Breakdancer^85^. Specifically, we first applied the packages LUMPY and Breakdancer to identify insertions, deletions, duplications, inversions and translocations for 1,081 accessions. The raw merged dataset contained insertions, deletions, inversions and duplications but not translocations. Next, each structural variation call was evaluated by local assembly using Velvet^86^, and then contig alignments were computationally parsed to determine if there was supporting evidence for the structural variation, and to localize the breakpoints of the structural variation. On the basis of the above pipeline, the above four kinds of structural variation call sets were obtained. For translocation, we considered the calls supported by both LUMPY and Breakdancer to be reliable. Finally, for the whole set, we merged the calls of all individuals to a nonredundant set and ensured that each call had at least ten accessions to support. We constructed the phylogenetic tree applying TreeBest software (v.1.9.2).

To identify NDM373-9 fragments transferred from *G.* *barbadense* Pima90, we separately mapped the resequences of NDM373-9 and CCRI8 to the NDM8 reference genome and detected the specific structural variations of NDM373-9. Finally, we obtained the overlapped structural variations by comparing these structural variations to the specific structural variations of Pima90 against the NDM8 genome.

### GWAS analysis

As we know, At08 possessed abundant inversions^6^ that might interfere with the accuracy of GWAS. Thus, we used 277,292 structural variations excluding those located on At08 and phenotypic data to perform GWAS for the seven traits, including FL, FS, M, BW, LP, SI and VW resistance.

For fiber quality and yield traits, we used the data in our previous research, 12 environments for the core collection of 419 accessions^8^ and eight environments for the 662 expanded accessions^36,87^. In addition, we newly obtained fiber quality trait data of 419 accessions collected from the Hainan breeding nursery in 2016 and 2017 and fiber quality and yield trait data for all the above 1,041 accessions from the Qingxian breeding nursery in 2019. The means and BLUP^88^ were used to perform GWAS. The BLUP was calculated with lme4 packages (1.1–23) in R (v.3.6.3), and the formula was as follows:

$${{Y}} = \mu + {\mathrm{Line}} + {\mathrm{Loc}} + \left( {{\mathrm{Line}} \times {\mathrm{Loc}}} \right) + \left( {{\mathrm{Rep}} \times {\mathrm{Loc}}} \right) + \varepsilon$$

where *Y*, *μ*, Line and Loc represent phenotype, intercept, variety effects and environmental effects, respectively. Rep means different repetitions and *ε* represents random effects. Line × Loc represents the interaction between variety and environment, and Rep × Loc represents the interaction between repetition and environment.

For VW resistance evaluation, we used the high-pathogenicity strain LX2-1 to inoculate 401 out of 1,081 accessions. For each accession, we performed four independent experiments in growth chamber; 35 seedlings were analyzed in each experiment for each accession. The susceptible variety Jimian11 and the resistant variety ND601 were used as controls to monitor the accuracy of disease determination. Symptom development was recorded at 20 days post inoculation (dpi) and categorized into five grades recorded as 0 to 4. The DI was calculated according to a previous method^37^.

Association analysis was conducted with the genome-wide efficient mixed-model association (GEMMA) software package^89^. The top three principal components (PCs) were used to build up the S matrix for population–structure correction. The matrix of simple matching coefficients was used to build up the K matrix. The genome-wide significance threshold was set as *P* = 1/*n* (*n*, total number of structural variations).

### RNA extraction and qRT–PCR analysis

Total RNA was extracted using the EASYspin Plus Plant RNA Kit (Aidlab Biotech) according to the manufacturer’s protocols. cDNA was generated with a PrimeScript RT Reagent Kit with gDNA Eraser (TaKaRa). We performed qRT–PCR with a SYBR Premix DimerEraser (Perfect Real Time) (TaKaRa). *Ghhistone3b* was used to normalize all qRT–PCR data. The relative expression was calculated using the 2^−ΔΔCt^ method^90^. The primers used for gene expression analysis were listed in Supplementary Table 60.

### Generation of transgenic *Arabidopsis* and disease assays

For *GhM_D11G3743* overexpression, full-length open reading frame was amplified by PCR using cDNA synthesized from RNA that was isolated from seedlings of NDM8. The amplified product was further cloned into the pGreen vector under the control of the cauliflower mosaic virus 35S promoter. The transformed seedlings were identified on the basis of Basta screening and PCR detection. T_3_ seeds of transgenic lines were used for phenotypic analyses. *Arabidopsis* plants (20 d old) were inoculated with Vd as previously described^37^. Disease development was monitored for up to 28 dpi and DI was calculated according to a previous description^37^.

### Virus-induced gene silencing in cotton and pathogen inoculation

The gene-specific region for *GhM_D11G3743* (*GhNCS*) was amplified as a template and cloned into the pTRV2 vector. The resulting pTRV2 construct was coinfiltrated with pTRV1 via *Agrobacterium tumefaciens* GV3101 into cotton seedlings of resistant NDM8 and susceptible CCRI8, through syringe inoculation when the cotyledons opened^91^. Plants coinfiltrated with empty pTRV2 and pTRV1 were used as mock controls. After 2 weeks, the plants were inoculated with a Vd spore suspension (around 1 × 10^7^ conidia per milliliter). We performed the experiments with at least 35 seedlings per treatment and repeated them twice. We determined the silent efficiency of cotton by using mix sample with all the treated seedlings. The DI was calculated as above. Primers used for construction of a VIGS vector are listed in Supplementary Table 60.

### Statistical analysis

We performed permutation tests 1,000,000 times on the basis of the count density of the structural variations through dividing each chromosome into 1,000 sliding windows. The Mann–Whitney *U*-test was used to perform a statistical analysis on the densities of structural variations and LTRs. SPSS22 was used for statistical analysis of the phenotypic traits. We performed one-way analysis of variance, and the significance level was set at *P* = 0.05 or 0.01. In transcriptome analyses, the RPKM values of genes from each sample were calculated with Cufflinks (v.2.1.1)^66^. Two-tailed Student’s *t-*tests were used to compare *GhNCS* expression levels between resistant and susceptible varieties, the DI values of the silent and mock plants and the DI values of overexpression *Arabidopsis* and mock plants.

### Reporting Summary

Further information on research design is available in the Nature Research Reporting Summary linked to this article.

## Online content

Any methods, additional references, Nature Research reporting summaries, source data, extended data, supplementary information, acknowledgements, peer review information; details of author contributions and competing interests; and statements of data and code availability are available at 10.1038/s41588-021-00910-2.

## Supplementary information

Supplementary Information Supplementary Figs. 1–12

Reporting Summary

Supplementary Table 1

Supplementary Data 1

Supplementary Data 2

Supplementary Data 3

## Data Availability

The raw sequencing data and transcriptome data of NDM8 and Pima90, and the resequencing data of 1,081 accessions are deposited in the NCBI Sequence Read Archive under the BioProject accession number PRJNA680449. The two cotton assemblies have been deposited in NCBI GenBank under the accession numbers JAHMMW000000000 and JAHMMX000000000. The versions described in this paper are version JAHMMW000000000.1 and JAHMMX000000000.1. The relevant data are also deposited in the CottonGen database https://www.cottongen.org/ (the assemblies and gene annotations) and are available at the website http://cotton.hebau.edu.cn/Data%20Download.html (the assemblies, gene annotations, structural variations and phenotypic data).

## Code availability

Code used for the genome annotations of repetitive elements and gene structures are deposited in Zenodo DOI-minting repository with the 10.5281/zenodo.4851529.
