# Supplementary material for: High-quality genome assembly and resequencing of modern cotton cultivars provide resources for crop improvement
Source: Nat Genet. 2021 Aug 9;53(9):1385–91. doi: 10.1038/s41588-021-00910-2 (PMC8423627; doi:10.1038/s41588-021-00910-2)
Supplement: Supplementary file 1 — Supplementary Figs. 1–12 [file 41588_2021_910_MOESM1_ESM.pdf]

nature portfolio

https://doi.org/10.1038/s41588-021-00910-2

Supplementary information

High-quality genome assembly and resequencing of modern cotton cultivars provide resources for crop improvement

In the format provided by the authors and unedited

High-quality genome assembly and resequencing of modern cotton cultivars provide resources for crop improvement

Supplementary information

Table of Contents

Supplementary Figures 1-12

Table of contents:

Supplementary item	Title	Page
Supplementary Figure 1	Collinearity comparisons of the genomes between NDM8 and TM-1_HAU, and between Pima90 and 3-79_HAU	1
Supplementary Figure 2	Comparison of *Copia* and *Gypsy* number per Mb in NDM8 and Pima90 genomes	2
Supplementary Figure 3	Genomic landscape of Pima90 and NDM8 genomes	3
Supplementary Figure 4	Comparison of insertion and deletion number per Mb between At and Dt in Pima90	4
Supplementary Figure 5	Characterization of the inversions in NDM8 versus TM-1_HAU and Pima90 versus NDM8	5
Supplementary Figure 6	Pedigree of *G. hirsutum* line NDM373-9	6
Supplementary Figure 7	Pedigree and agronomic traits of *G. hirsutum* NDM8 and TM-1	7
Supplementary Figure 8	Comparison of insertion and deletion number per Mb between At and Dt in NDM8	8
Supplementary Figure 9	Comparison of the inversion number per Mb between At and Dt in NDM8	9
Supplementary Figure 10	Distribution of the identical ordered synteny blocks compared NDM8 to TM-1_HAU genome	10
Supplementary Figure 11	Phylogenetic tree of 1,081 cotton accessions based on 2,970,970 SNPs	11
Supplementary Figure 12	Genome-wide average linkage disequilibrium (LD) decay estimated using SNPs from 1,081 accessions	12

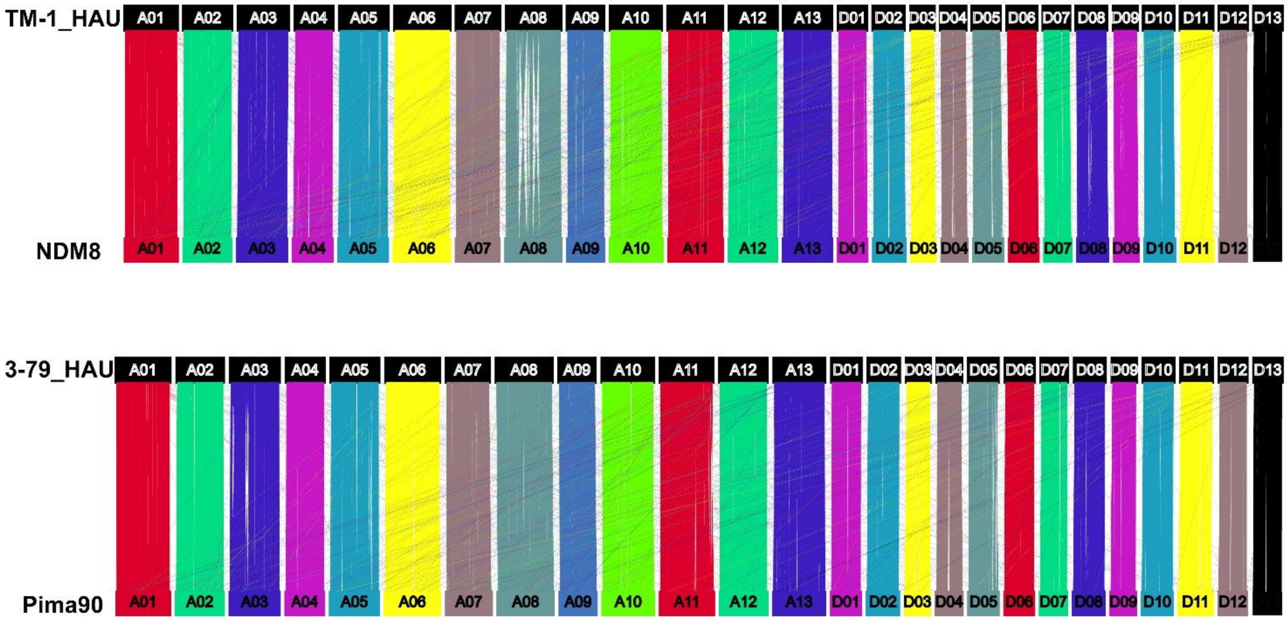

Supplementary Fig. 1 Collinearity comparisons of the genomes between *G. hirsutum* NDM8 and TM-1_HAU (top), and between *G. barbadense* Pima90 and 3-79_HAU (bottom).

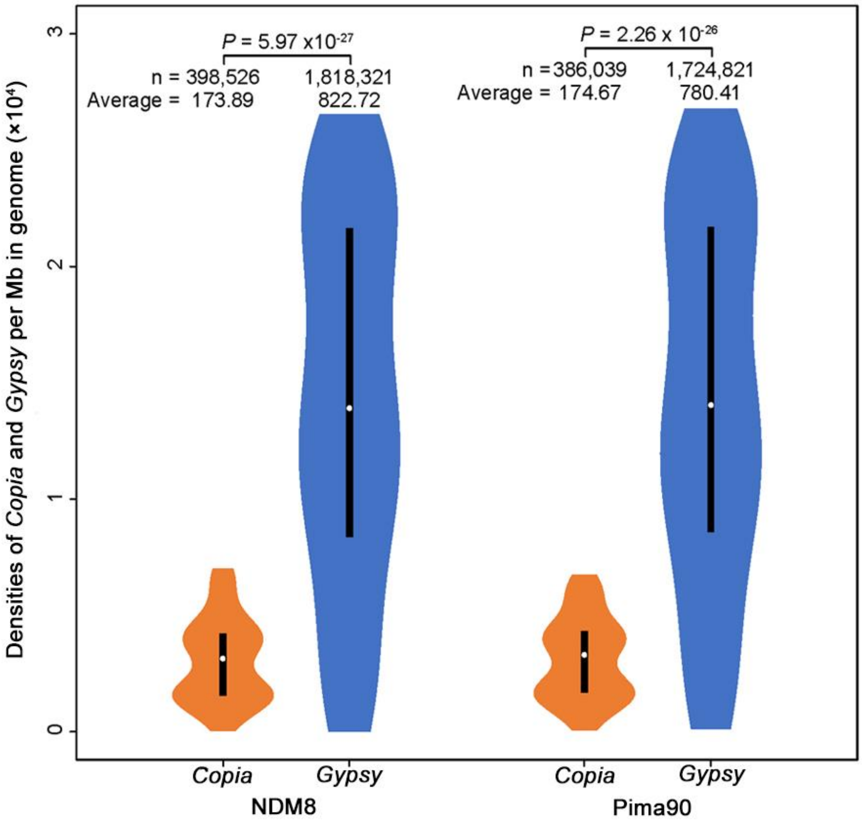

Supplementary Fig. 2 Comparison of *Copia* and *Gypsy* number per Mb in NDM8 and Pima90 genomes.

We determined the statistical significance using two-tailed Mann-Whitney *U* test. "n" means the number of *Copia* and *Gypsy* in NDM8 and Pima90. "Average" represents an average number of *Copia* and *Gypsy* per Mb along the allotetraploid cotton genomes. Data are represented as mean values ± s.d. Violin shape, *Copia* and *Gypsy* density curves; white node in center, median; black box inside the violin, box-and-whisker plot.

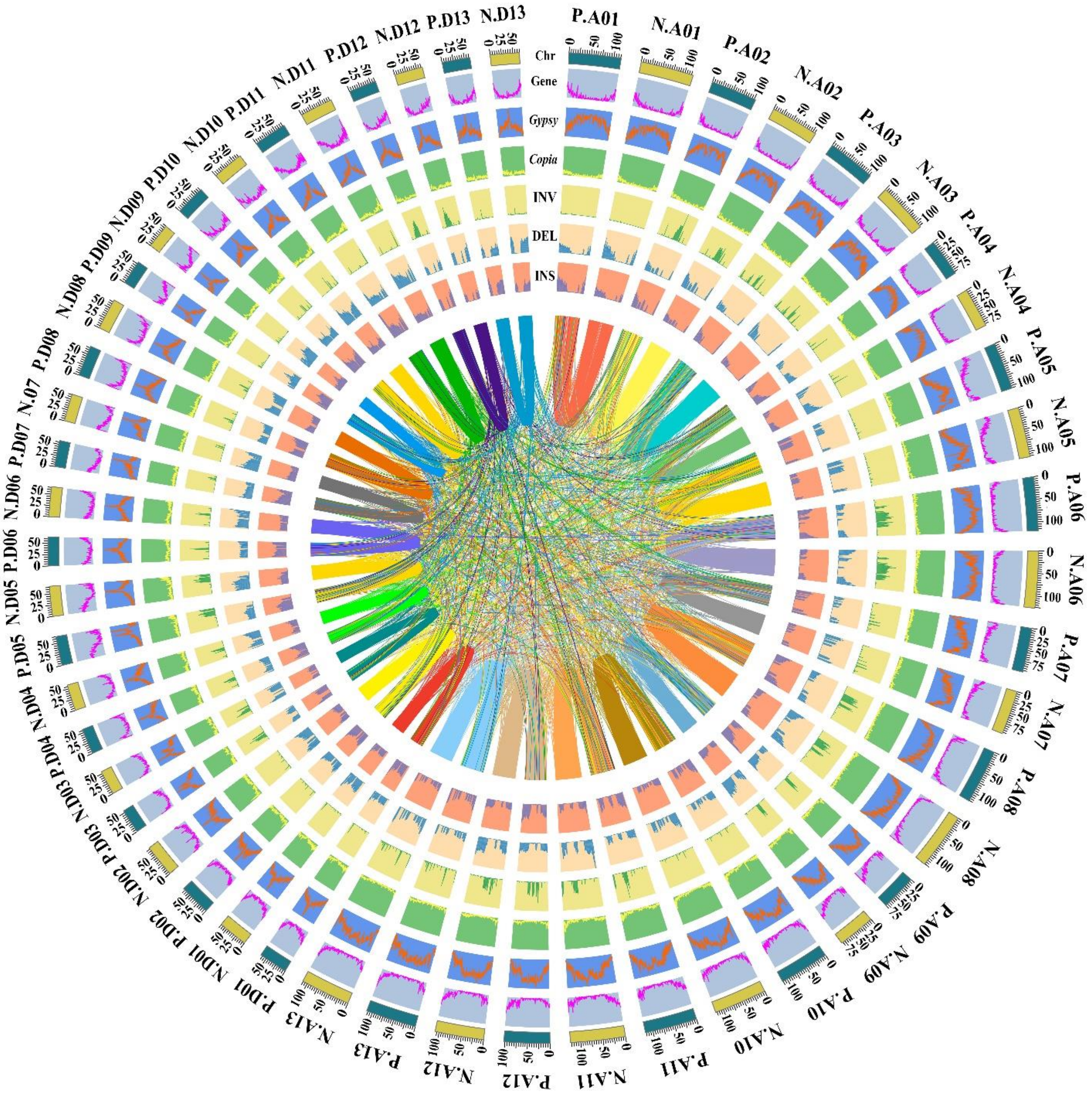

Supplementary Fig. 3 Genomic landscape of Pima90 and NDM8 genomes.

P.A01 to P.D13 and N.A01 to N.D13 represent the chromosomes of Pima90 and NDM8, respectively. **Chr**, Length of chromosome (Mb). **Gene**, Gene length distribution. ***Gypsy***, *Gypsy* length distribution. ***Copia***, *Copia* length distribution. **INV**, Density distribution of inversion. **DEL**, Density distribution of deletion. **INS**, Density distribution of insertion. The inner circle indicates the synteny between the two genomes. The sliding windows are non-overlapped with a 500-kb length.

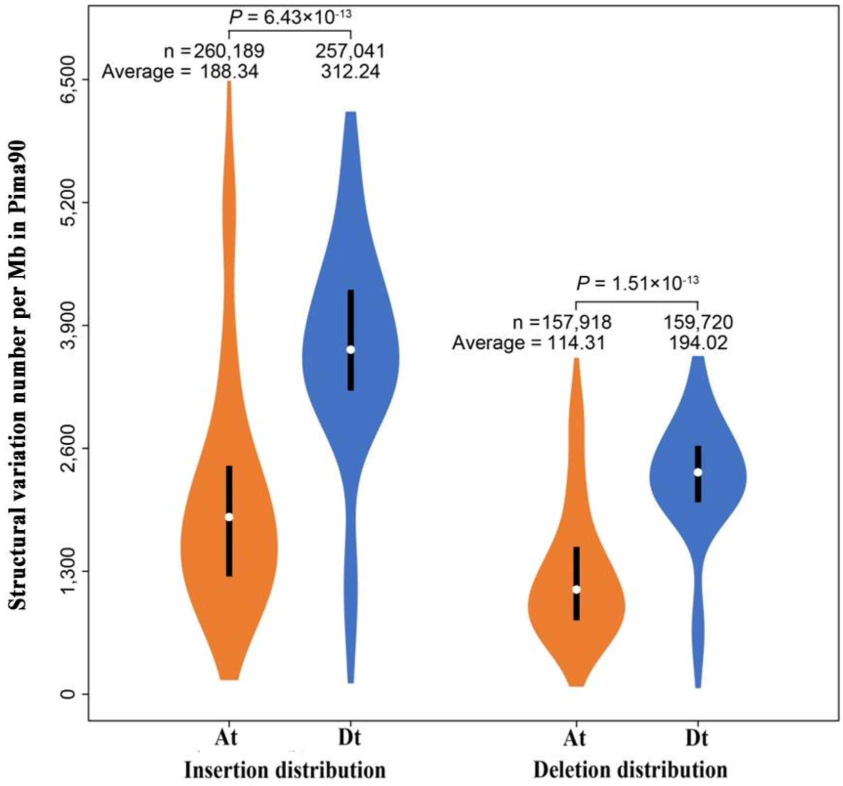

Supplementary Fig. 4 Comparison of insertion and deletion number per Mb between At and Dt in Pima90.

We determined the statistical significance using two-tailed Mann-Whitney *U* test. "n" means the number of structural variations in At and Dt. "Average" represents an average number of structural variations per Mb along the Pima90 genomes. Data are represented as mean values ± s.d. Violin shape, structural variations density curves; white node in center, median; black box inside the violin, box-and-whisker plot.

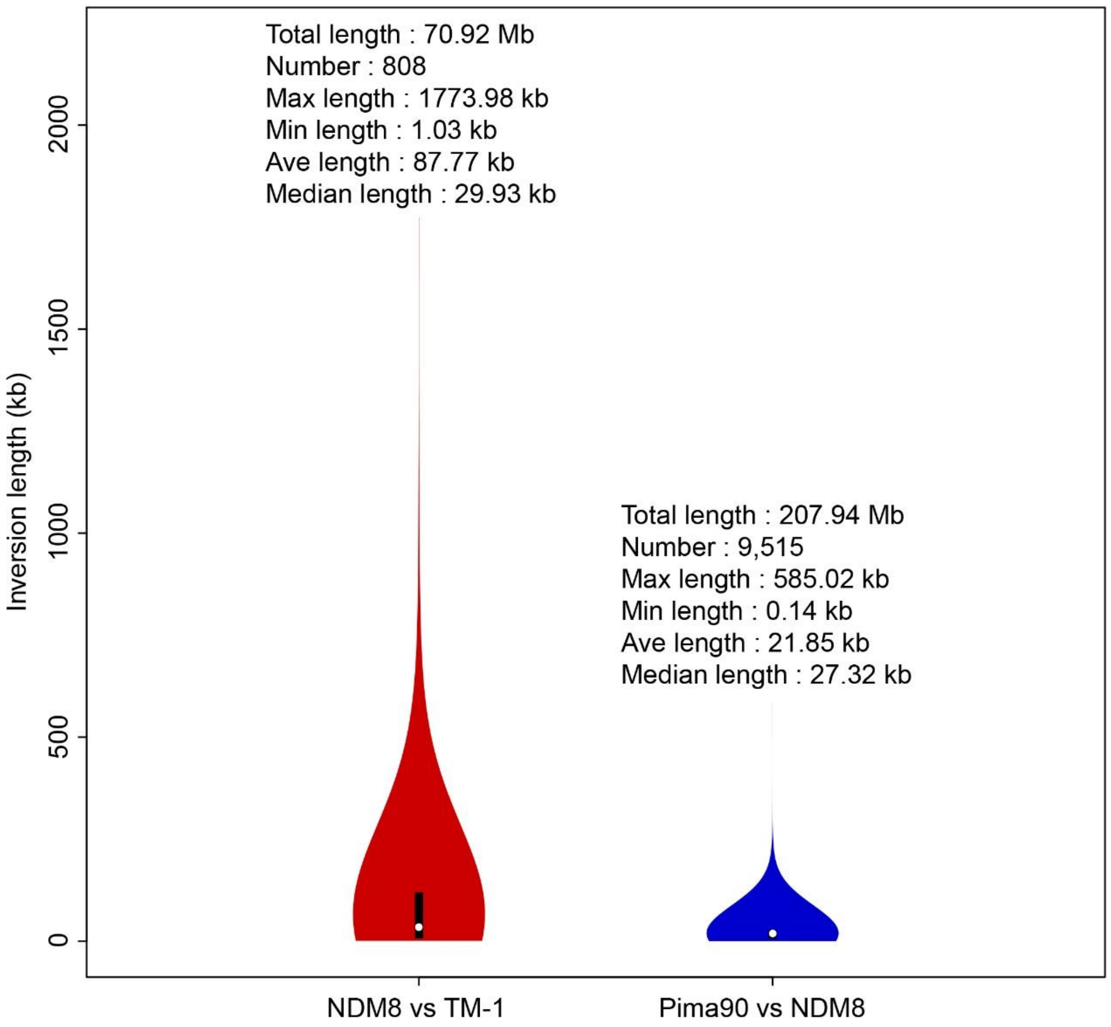

Supplementary Fig. 5 Characterization of the inversions in NDM8 versus TM-1_HAU and Pima90 versus NDM8.

Data are represented as mean values ± s.d. Violin shape, inversion length density curves; white node in center, median; black box inside the violin, box-and-whisker plot.

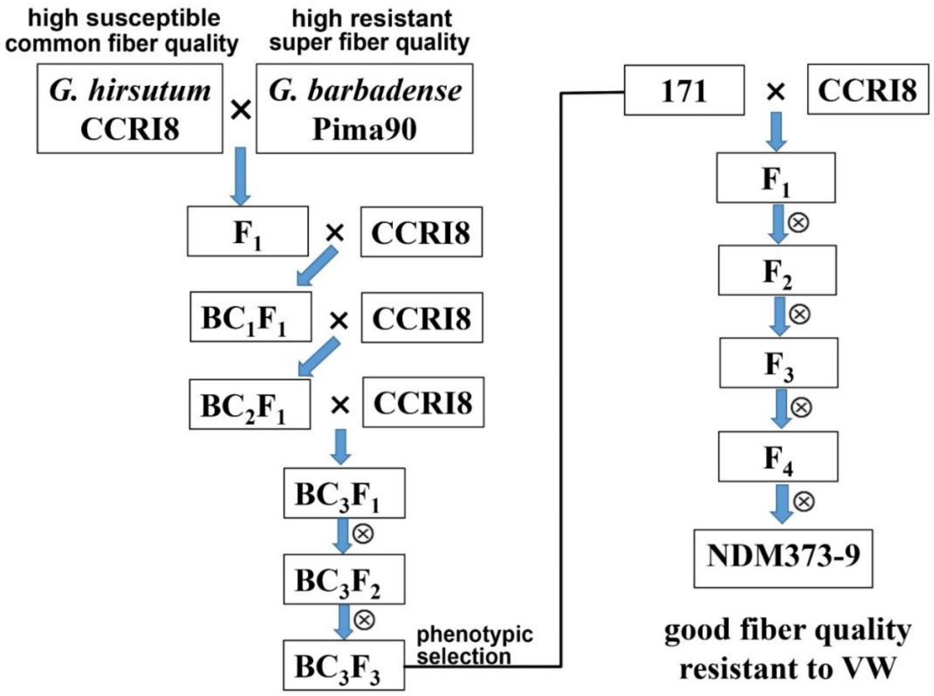

Supplementary Fig. 6 Pedigree of *G. hirsutum* line NDM373-9.

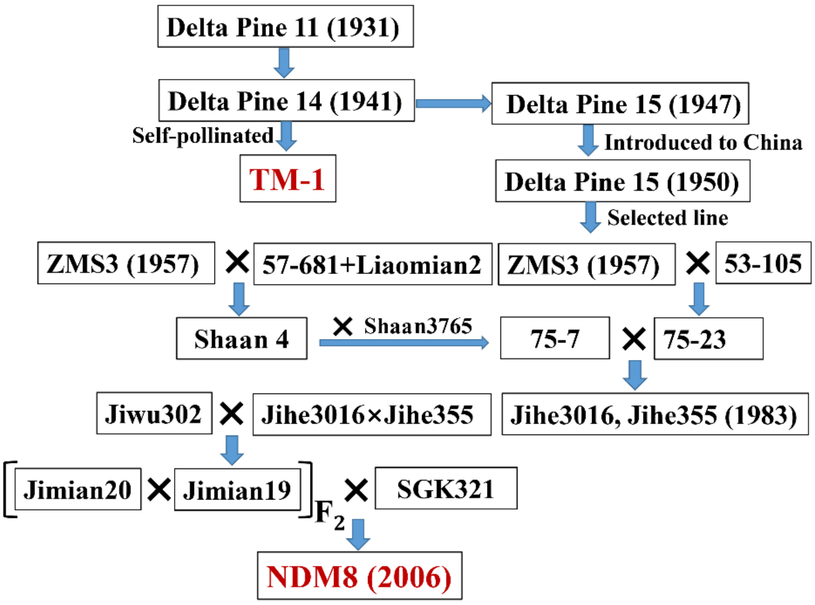

Variety	Fiber length (mm)	Fiber strength (cN/tex)	Micronaire value	Boll weight (g)	Lint percentage (%)	VW resistance
TM-1	29.03	27.15	4.58	5.83	34.03	susceptible
NDM8	30.50	30.40	4.60	5.90	40.50	resistant

Supplementary Fig. 7 Pedigree and agronomic traits of *G. hirsutum* NDM8 and TM-1.

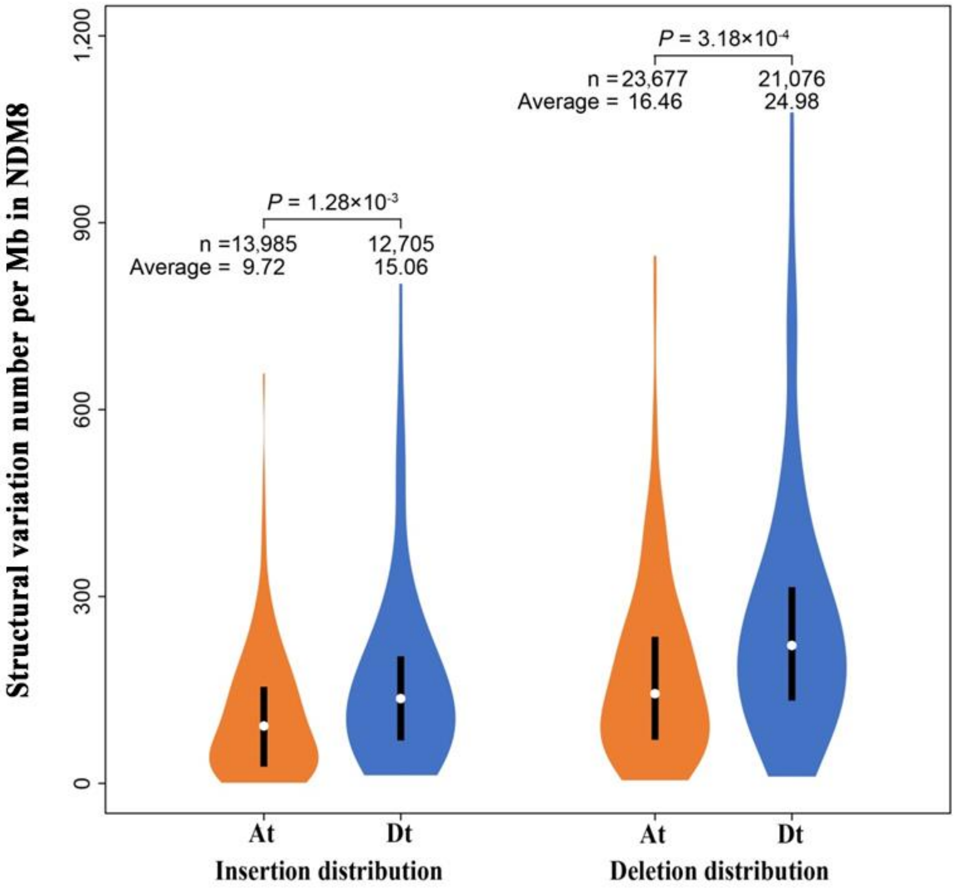

Supplementary Fig. 8 Comparison of insertion and deletion number per Mb between At and Dt in NDM8.

We determined the statistical significance using two-tailed Mann-Whitney *U* test. "n" means the number of structural variations in At and Dt. "Average" represents an average number of structural variations per Mb along the NDM8 genomes. Data are represented as mean values ± s.d. Violin shape, structural variations density curves; white node in center, median; black box inside the violin, box-and-whisker plot.

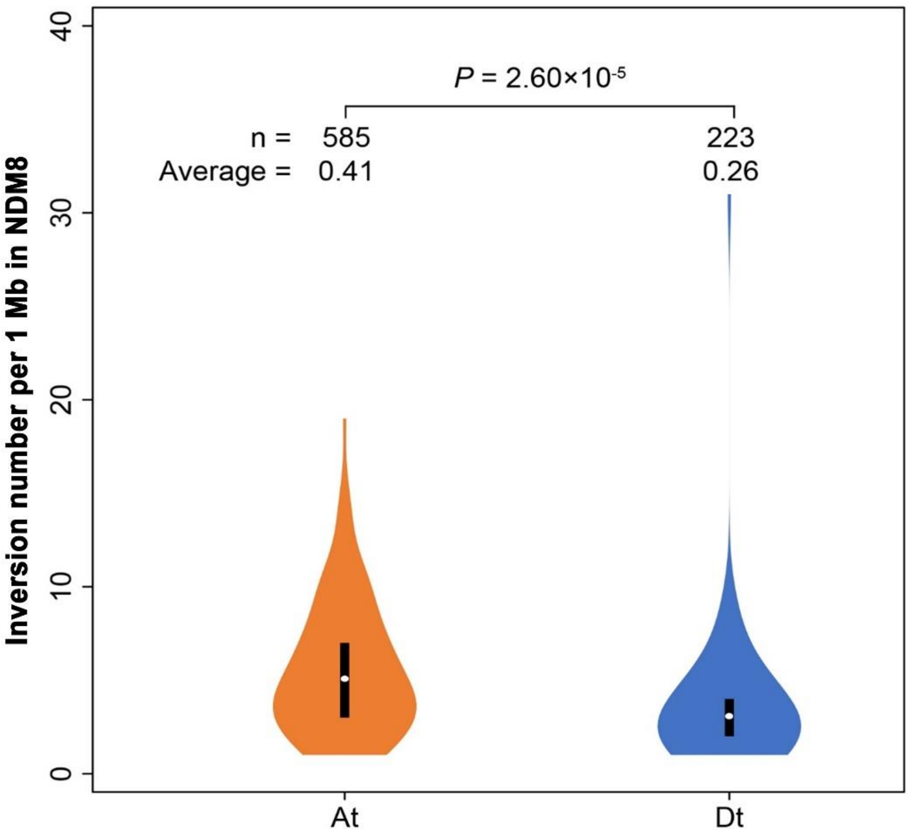

Supplementary Fig. 9 Comparison of the inversion number per Mb between At and Dt in NDM8.

We determined the statistical significance using two-tailed Mann-Whitney *U* test. "n" means the number of inversions in At and Dt of NDM8 genome. "Average" represents an average number of inversions per Mb along the At and Dt genomes. Data are represented as mean values ± s.d. Violin shape, inversion density curves; white node in center, median; black box inside the violin, box-and-whisker plot.

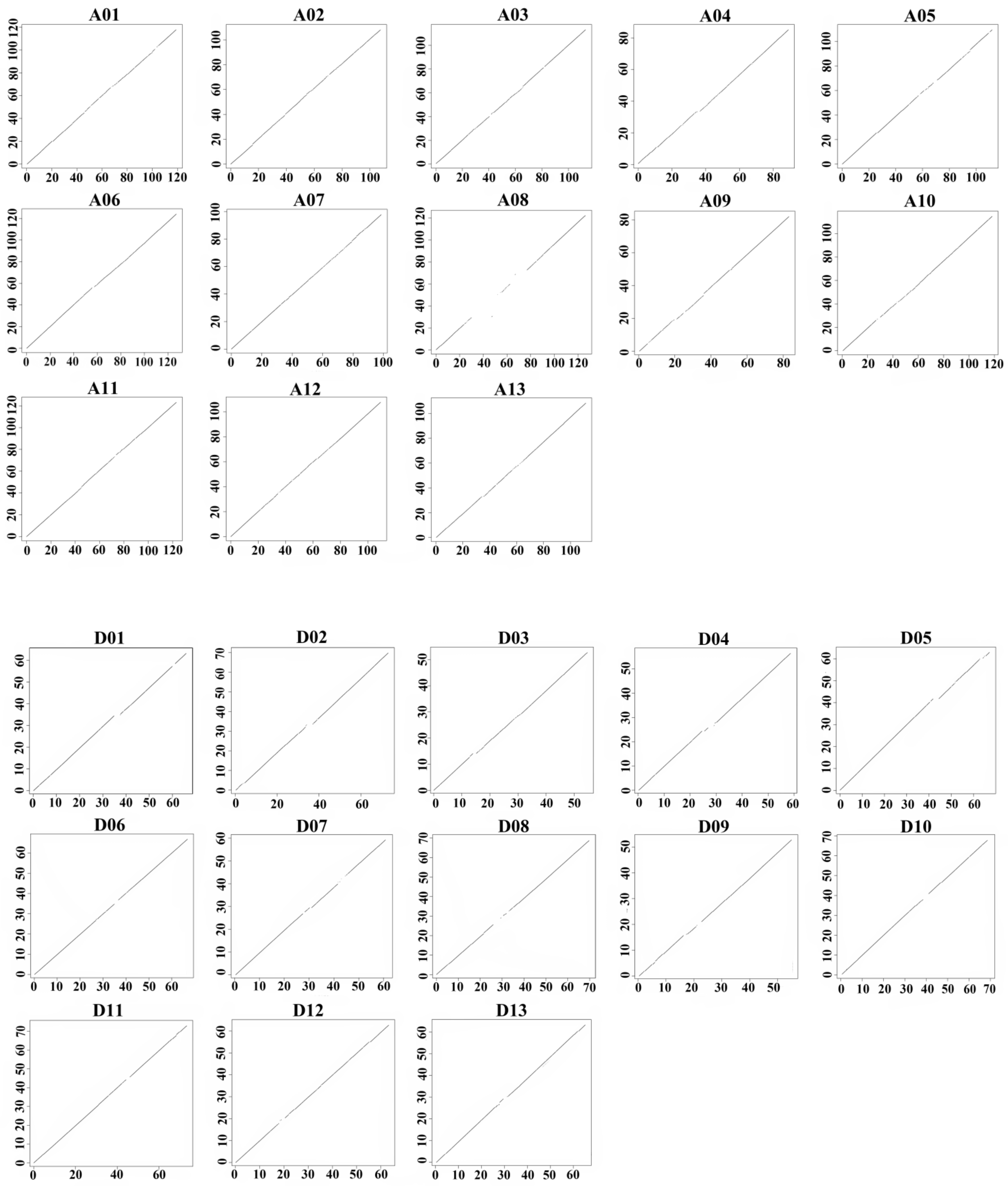

Supplementary Fig. 10 Distribution of the identical ordered synteny blocks compared NDM8 to TM-1_HAU genome.

The horizontal and vertical axes represent TM-1_HAU chromosome and NDM8, respectively.

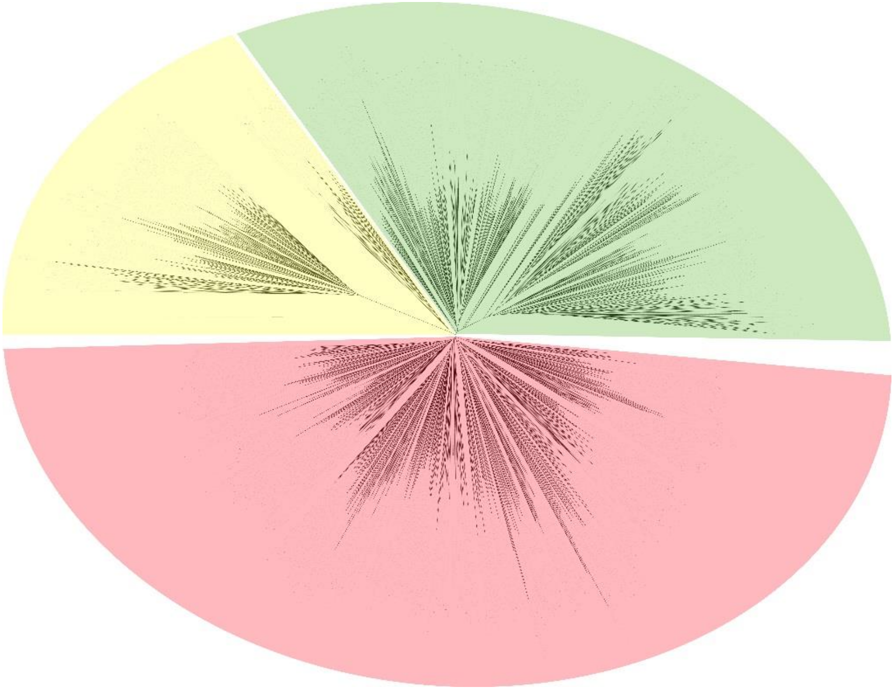

Supplementary Fig. 11 Phylogenetic tree of 1,081 cotton accessions based on 2,970,970 SNPs. The green, red and yellow indicate group I, II and III, respectively. Group I represented by modern-time variety Lumianyan28; Group II represented by early-time variety Deltapine15; Group III represented by intermediate-time variety Jimian8.

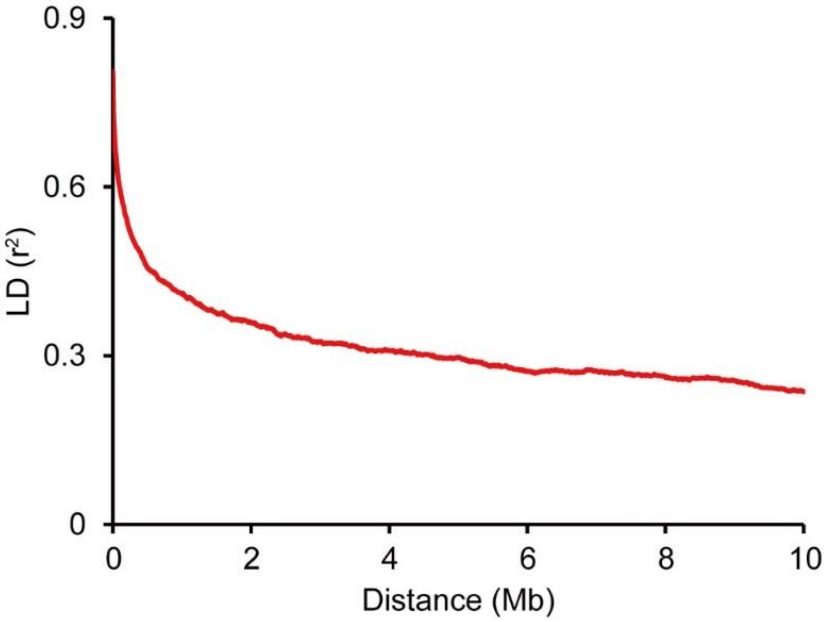

Supplementary Fig. 12 Genome-wide average linkage disequilibrium (LD) decay estimated using SNPs from 1,081 accessions.

LD decay value is 325 kb when r^2 declines to 0.5, and 1.1 Mb when r^2 declines to a half, respectively.
